# *Telipogon marietalamae* and *T. puyensis* (Orchidaceae, Oncidiinae), two new species from the Amazonian region of Ecuador

**DOI:** 10.3897/phytokeys.280.196965

**Published:** 2026-10-22

**Authors:** Marco M. Jiménez, Nadia Lapo-Gonzalez, Diego Gutiérrez del Pozo, J. R. Kuethe, Luis Baquero, Henry X. Garzón-Suárez, Carlos Martel, Gabriel A. Iturralde

**Affiliations:** 1 Grupo de Investigación en Biodiversidad, Medio Ambiente y Salud (BIOMAS), Carrera de Ingeniería en Agroindustria, Facultad de Ingenierías y Ciencias Aplicadas, Universidad de Las Américas, UDLA, Vía a Nayón, Quito 170124, Ecuador Grupo de Investigación en Biodiversidad, Medio Ambiente y Salud (BIOMAS), Carrera de Ingeniería en Agroindustria, Facultad de Ingenierías y Ciencias Aplicadas, Universidad de Las Américas, UDLA Quito Ecuador; 2 Floplaya, Compañía de Flores y Plantas Yantzaza S.A., El Pangui, 190650, Ecuador Floplaya, Compañía de Flores y Plantas Yantzaza S.A. El Pangui Ecuador; 3 Grupo Científico Calaway Dodson: Investigación y Conservación de Orquídeas del Ecuador, Quito, 170510, Pichincha, Ecuador Grupo Científico Calaway Dodson: Investigación y Conservación de Orquídeas del Ecuador Quito Ecuador; 4 Universidad Estatal Amazónica UEA, Herbario Amazónico del Ecuador ECUAMZ, Carretera Tena a Puyo Km. 44, Napo, Carlos Julio Arosemena Tola 150950, Ecuador Universidad Estatal Amazónica UEA, Herbario Amazónico del Ecuador ECUAMZ Carlos Julio Arosemena Tola Ecuador; 5 School of Environment, Faculty of Sciences, University of Auckland, City Campus for Science and Engineering, Symonds Street, Auckland, New Zealand School of Environment, Faculty of Sciences, University of Auckland, City Campus for Science and Engineering Auckland New Zealand; 6 Herbario HUTPL, Departamento de Ciencias Biológicas, Universidad Técnica Particular de Loja, Loja, Ecuador Herbario HUTPL, Departamento de Ciencias Biológicas, Universidad Técnica Particular de Loja Loja Ecuador; 7 Trait Diversity and Function, Royal Botanic Gardens, Kew, Richmond, TW9 3AB, UK Trait Diversity and Function, Royal Botanic Gardens, Kew Richmond United Kingdom

**Keywords:** Amazonian piedmont, Andean foothills, anthropogenic activities, *matK*, nrITS, Pastaza, secondary forest, synonym, *Telipogon leisberthvelezii*, Zamora Chinchipe

## Abstract

Two new species of miniature *Telipogon* (Orchidaceae: Oncidiinae) from eastern Ecuador, *Telipogon marietalamae* and *T. puyensis*, are described and illustrated. Phylogenetic analyses based on nuclear nrITS and plastid *matK* markers placed these two new taxa in distinct phylogenetic clades. Morphologically, the new species are distinguished from closely related taxa by unique combinations of floral characters. *Telipogon marietalamae* is characterized by its 1-veined ligulate-lanceolate petals, a velvety, basally swollen lip, and dense tufts of stellate column setae with few apical tips, whereas *T. puyensis* is characterized by a facultatively branched inflorescence and column setae that are hispid from near the middle to the apex. The new species inhabit disturbed or secondary forests and are restricted to the Amazonian piedmont and Andean foothills of Ecuador. Furthermore, *T. reventadorensis* is treated here as a heterotypic synonym of *T. hystrix*, based on studies of field populations, and a taxonomic key for all known Ecuadorian miniature *Telipogon* is generated. These findings highlight the underestimated diversity of miniature *Telipogon* in the Andean–Amazonian region of Ecuador.

## Introduction

*Telipogon* Kunth is a species-rich Neotropical genus of Orchidaceae, currently comprising approximately 260–280 species (Martel et al. 2025; POWO 2026). Its distribution ranges from southern Mexico to Panama in Central America, the Dominican Republic in the Caribbean, and across northern and western South America, including Venezuela, Colombia, Ecuador, Peru, and Bolivia, with its center of diversity in the northern Andes, particularly in Colombia and Ecuador (Chase 2009; Pupulin 2022).

The genus is characterized by a highly distinctive floral morphology, including usually non-resupinate flowers that are often showy and patterned with stripes or spots, a short column frequently bearing tufts of prominent setae, and a pollinarium with an uncinate viscidium (Chase 2009; Martel et al. 2020). While many species display relatively large and visually striking flowers, a subset of taxa is characterized by small plants (often less than 10 cm tall) and flowers usually less than 2 cm in diameter. These taxa were historically classified under different genera (i.e., *Cordanthera* L.O.Williams, *Darwiniera* Braas & Lückel, *Dipterostele* Schltr., *Sodiroella* Schltr., and *Stellilabium* Schltr.; Schlechter 1915, 1921; Braas and Lückel 1982; Garay and Romero 1998), some of which were later treated as sections within an expanded *Stellilabium* (Dressler 1999). However, molecular phylogenetic studies revealed that species assigned to the expanded concept of *Stellilabium* were nested within *Telipogon* and did not form a monophyletic group (Williams et al. 2005; Neubig et al. 2012), leading to their transfer to the latter (Williams et al. 2005). The term “miniature *Telipogon*” has been proposed to refer to the former *Stellilabium* species (Martel et al. 2017).

Ecuador is one of the most diverse countries in terms of miniature *Telipogon*. Dodson (2004) listed 11 species treated as *Stellilabium* in his checklist of Ecuadorian orchids: *Telipogon alexii* N.H.Williams & Dressler, *T. alticola* (Dodson & R.Escobar) N.H.Williams & Dressler, *T. astroglossus* Rchb.f., *T. embreei* N.H.Williams & Dressler, *T. hystrix* (Dodson) N.H.Williams & Dressler, *T. jostii* (Dodson) N.H.Williams & Dressler, *T. microglossus* (Schltr.) N.H.Williams & Dressler, *T. morganiae* (Dodson) N.H.Williams & Dressler, *T. pogonostalix* Rchb.f., *T. reventadorensis* N.H.Williams & Dressler, and *T. williamsii* P.Ortiz. Five additional species were added in subsequent years (Szlachetko and Margońska 2006; Kolanowska et al. 2017; Zambrano et al. 2018; Jiménez et al. 2024; Iturralde et al. 2024; Jiménez et al. 2025): *T. leisberthvelezii* Iturralde, M.M.Jiménez & H.Garzón, *T. nigropurpureus* P.Ortiz, *T. sonia-juaniorum* Zambrano, Bogarín & Solano, *T. villonacoensis* M.M.Jiménez, Iturralde & C.Martel, and *T. amicorum* (Szlach. & Marg.) J.M.H.Shaw, the latter synonymized with *T. astroglossus* (Martel 2016). At present, 16 species of miniature *Telipogon* are recognized for Ecuador, including the taxonomic adjustments proposed herein (see key to species of miniature *Telipogon* at the end of this document).

Miniature *Telipogon* have been under-collected due to their small size and inconspicuous habit. As a result, their diversity, distribution, and taxonomy are still incompletely known. Recent fieldwork conducted in the Amazonian region of eastern Ecuador has led to the discovery of two new species. In addition, a critical reassessment of previously described eastern Ecuadorian taxa supports a nomenclatural adjustment within the group. Based on morphological and genetic analyses, this study describes and illustrates two new species of *Telipogon*, proposes the synonymization of *T. reventadorensis* under *T. hystrix*, and produces a taxonomic key for all known Ecuadorian miniature *Telipogon*.

## Materials and methods

### Study area, fieldwork, and sampling

Fieldwork took place in eastern Ecuador. One *Telipogon* species was discovered by one of the authors (NLG) near Pachicutza, Zamora Chinchipe Province, southeastern Ecuador, during a botanical inventory survey in January 2023. Subsequent field explorations in the same area in 2026 led to the discovery of a second population located approximately 2.3 km from the first locality.

Another taxonomic novelty was discovered in the surroundings of the ecotourism trail along the Puyo River, near the city of Puyo, Pastaza Province, central-eastern Ecuador. One of the authors (DGP) first encountered a flowering individual at this locality in 2016. In 2024, targeted field explorations were conducted in the same area, resulting in the discovery of a small patch consisting of three individuals, as well as an additional individual located approximately 500 m from the first locality.

Targeted expeditions were also carried out to type localities and nearby areas of taxa previously described from northeastern Ecuador. These surveys allowed the examination of populations attributed to *Telipogon hystrix* near the localities of Mera (Pastaza Province) and Topo (Tungurahua Province), as well as material referable to *T. reventadorensis* near Borja, Napo Province.

Voucher specimens were collected and pressed following standard herbarium techniques. Flowers were preserved in a solution of 70% ethanol, 29% water, and 1% glycerol for detailed morphological study. Leaf samples intended for subsequent molecular analyses were dried and stored in silica gel. Vegetative and floral structures were documented *in situ* and *ex situ* using a Panasonic FZ300 camera equipped with a Raynox DCR-250 super macro lens and a Nikon D3500 camera with an AF-S DX Micro Nikkor 40 mm f/2.8G lens. Photographs were taken *in situ* to document habitat, plant habit, and flower morphology; detailed photographs of plants and entire and dissected flowers were taken *ex situ* using a black suede cloth with a reference scale, allowing precise measurements using the open-source software ImageJ 1.53m (https://ij.imjoy.io/). Photographs were then used to prepare figures, which were edited using Adobe Photoshop® 2019 (San Jose, CA, USA). The ecosystem types in which the species occur were identified and classified using the official Ecuadorian Ecosystem Classification System (Ministerio del Ambiente 2013; MAE 2026). Collected vouchers were deposited in the Herbarium of the Universidad Técnica Particular de Loja (HUTPL) and the Herbario del Museo Ecuatoriano de Ciencias Naturales (QCNE). Field collections were carried out under research permits issued by the Ministerio del Ambiente y Transición Ecológica of Ecuador (permit numbers MAATE-DNB-CM-2022-0248-M-0001 and MAATE-DBI-CM-2025-0458).

### Morphological analysis and comparative study

Morphological observations and measurements were conducted on both fresh and alcohol-preserved material, and an extensive revision of the described miniature *Telipogon* species was carried out. Available holotypes and type images were examined through online platforms, including Tropicos (https://www.tropicos.org/), iDigBio (https://portal.idigbio.org/), JSTOR Global Plants (https://plants.jstor.org/), and GBIF (https://www.gbif.org/). Protologues of all species described within this group were carefully reviewed and compared with the newly collected material (e.g., Reichenbach 1858; Dodson and Dodson 1980, 1982, 1984; Dressler 1999, 2001; Dodson 2004; Bogarín 2012; Martel et al. 2017; Jiménez et al. 2024). Additionally, herbarium specimens of morphologically similar species were examined, either physically or digitally, from the following herbaria: QCA, QCNE, and K (physical collections), and AMES, FLAS, MO, and SELBY (digital collections). The data obtained were used to prepare species descriptions, diagnoses, and diagnostic characters distinguishing the species from their most morphologically similar taxa. Terminology for vegetative and floral structures follows Beentje (2016).

### Distribution mapping and conservation assessment

To assess potential undocumented records, photographic databases and citizen science platforms, including iNaturalist and Flickr, were also surveyed. The distribution map was created using ArcGIS Desktop 10.3 (ESRI 2014). The extent of occurrence (EOO) and area of occupancy (AOO) were calculated using GeoCAT (https://geocat.iucnredlist.org/; Bachman et al. 2011), following the criteria and thresholds of the IUCN Red List Categories and Criteria (IUCN 2024), and were used as a basis for the preliminary conservation assessment.

### Phylogenetic analysis

Genomic DNA was isolated from silica-dried leaf material following a rapid extraction protocol *sensu*Kasajima et al. (2004) at the research laboratories of the Universidad de Las Américas (UDLA). Two molecular markers were selected for phylogenetic inference: the nuclear ribosomal internal transcribed spacer (nrITS) and the plastid maturase K (*matK*). Polymerase chain reactions (PCR) were performed with 7.5 µL of GoTaq Green Master Mix 2× (Promega), 3 µL of genomic DNA, 7.5 µL of ultrapure water, and 0.75 µL of each primer to a final volume of 19.5 µL. Primer sequences and PCR cycling conditions were as described by Iturralde et al. (2023).

PCR products were purified and sequenced using Sanger sequencing on an ABI 3500xL Genetic Analyzer (Applied Biosystems). New sequences were generated for five Ecuadorian *Telipogon* species and deposited in GenBank (see Table 1). Additional nrITS and *matK* sequences representing Central and South American *Telipogon* species were retrieved from GenBank (https://www.ncbi.nlm.nih.gov/genbank/) (Table 1). All subsequent analyses, including sequence assembly, alignment, phylogenetic inference, and tree visualization, were conducted in Geneious Prime 2022.1 (Biomatters Ltd., Auckland, New Zealand; https://www.geneious.com/). Sequence alignments were performed using the default settings of the Geneious alignment plugin. Since the dataset was small, alignments were inspected visually, and terminal regions containing more than 50% gaps across sequences were trimmed. No ambiguously aligned regions were detected, making automated trimming unnecessary.

**Table 1.** Sampling and accession information for species used in this study.

**Taxon**	**Source**	**Voucher**	**Herbarium**	**nrITS**	***matK***
*Fernandezia sanguinea* (Lindl.) Garay & Dunst.	NCBI	–	–	FJ565526	FJ565009
*Hofmeisterella eumicroscopica* (Rchb.f.) Rchb.f.	NCBI	–	–	DQ315823	AF350589
*Telipogon acicularis* (Dressler) N.H.Williams & Dressler	NCBI	–	–	DQ315837	DQ315896
*Telipogon alexii* N.H.Williams & Dressler	NCBI	–	–	PV983249	PV988129
*Telipogon cf. alticola* (Dodson & R.Escobar) N.H.Williams & Dressler	NCBI	–	–	PV983250	PV988130
*Telipogon ariasii* Dodson & D.E.Benn.	NCBI	–	–	DQ315852	DQ315902
*Telipogon astroglossus* Rchb.f.	NCBI	–	–	PV983251	PV988131
*Telipogon boliviensis* (R.Vásquez & Dodson) N.H.Williams & Dressler	NCBI			DQ315839	–
*Telipogon bombiformis* Dressler	NCBI			DQ315854	FJ564866
*Telipogon bowmanii* Rchb.f. (synonym of *T. vargasii* C.Schweinf.)	NCBI	–	–	DQ315880	DQ315912
*Telipogon butcheri* Dodson & R.Escobar	NCBI			DQ315855	DQ315904
*Telipogon dalstromii* Dodson	NCBI	–	–	DQ315861	DQ315906
*Telipogon hausmannianus* Rchb.f.	NCBI			OR689558	OR689580
*Telipogon helleri* (L.O. Williams) N.H.Williams & Dressler	NCBI	–	–	MF962882	MF962888
*Telipogon hystrix* (Dodson) N.H.Williams & Dressler	NCBI			DQ315841	DQ315899
*Telipogon hystrix* (Dodson) N.H.Williams & Dressler	This study	MJ-1590	Photo Voucher	PZ725717	PZ727209
*Telipogon hystrix* (Dodson) N.H.Williams & Dressler	This study	GI-2410-7156	QCNE	PZ725718	PZ727210
*Telipogon hystrix* (Dodson) N.H.Williams & Dressler	This study	GI-2409-5331	QCNE	PZ725722	PZ727214
*Telipogon hystrix* (Dodson) N.H.Williams & Dressler	This study	GI-2503-7852	QCNE	PZ725723	PZ727215
*Telipogon jostii* (Dodson) N.H.Williams & Dressler	This study	GI-2410-7088	QCNE	PZ725719	PZ727211
*Telipogon leisberthvelezii* Iturralde, M.M.Jiménez & H.Garzón	This study	HG163	QCNE	PZ725720	PZ727212
*Telipogon maduroi* Dressler	NCBI	–	–	DQ315867	FJ564867
***Telipogon marietalamae* N.Lapo-Gonzalez, M.M.Jiménez & Iturralde**	**This study**	**M.M. Jiménez 2708**	**HUTPL**	**PZ725716**	**PZ727208**
*Telipogon nervosus* (L.) Druce	NCBI	–	–	DQ315870	DQ315907
*Telipogon octavioi* Dodson & R.Escobar	NCBI			OR689559	OR689579
*Telipogon pillaropatatensis* Iturralde, Monteros & Baquero	NCBI			OR689556	OR689582
*Telipogon pogonostalix* Rchb.f.	NCBI	–	–	AF239392	AF239488
*Telipogon pogonostalix* Rchb.f.	NCBI	–	–	PV983252	PV988132
*Telipogon pulcher* Rchb.f.	NCBI	–	–	DQ315875	DQ315910
***Telipogon puyensis* M.M.Jiménez, Iturralde & Baquero**	**This study**	**M.M. Jiménez et al. 2438**	**HUTPL**	**PZ725721**	**PZ727213**
*Telipogon smaragdinus* (Pupulin & M.A.Blanco) N.H.Williams & Dressler	NCBI			DQ315844	FJ565034
*Telipogon venustus* Schltr.	NCBI	–	–	FJ565183	FJ564703
*Telipogon villonacoensis* M.M.Jiménez, Iturralde & C.Martel	NCBI			PV983253	PV988133
*Telipogon williamsii* P.Ortiz	NCBI	–	–	PV983255	PV988135
*Trichoceros antennifer* (Bonpl.) Kunth	NCBI	–	–	DQ315883	FJ564953

The final dataset included 35 terminal taxa. *Hofmeisterella eumicroscopica* Rchb.f. and *Trichoceros antennifer* Kunth, both members of the *Telipogon* alliance (Williams et al. 2005; Martel et al. 2020), together with *Fernandezia sanguinea* (Lindl.) Garay & Dunst., were selected as outgroups (Table 1). The best-fit nucleotide substitution models for each marker were obtained in MEGA v12.1.2 (Tamura et al. 2021) using statistical model-selection criteria: GTR+G+I for nrITS and T92+I for *matK*. Phylogenetic relationships were inferred using Bayesian inference (BI) and maximum likelihood (ML). Both analyses were conducted on independent nrITS and *matK* datasets and on the concatenated dataset using gene partitions (nrITS: 1–624; *matK*: 625–1809), with each partition assigned its corresponding substitution model.

Bayesian inference was performed using the MrBayes 3.2.6 plugin (Huelsenbeck and Ronquist 2001), with two independent Markov chain Monte Carlo (MCMC) runs of four chains, each run for five million generations, sampling parameters and trees every 2,000 generations, and discarding the first 25% of samples as burn-in. Convergence was assessed by monitoring the average standard deviation of split frequencies (< 0.02), potential scale reduction factor (PSRF) values approaching 1.0, and effective sample sizes (ESS > 200). A 50% majority-rule consensus tree was generated, and Bayesian posterior probabilities were used as measures of nodal support.

Maximum likelihood analyses were conducted using RAxML v8.2.11 (Stamatakis 2014). Independent ML analyses of the nrITS, *matK*, and partitioned concatenated datasets were performed under the GTR+Γ model, with all model parameters estimated during tree search. Although preliminary model-selection tests identified different best-fit substitution models for the individual markers, RAxML exclusively implements the GTR model family for nucleotide data. Consequently, the GTR+Γ model was applied to both partitions. Nodal support was assessed using the rapid bootstrap algorithm with 1,000 replicates, and bootstrap values were mapped onto the best-scoring ML topology.

Resulting trees were visualized and annotated, and ML bootstrap values and Bayesian posterior probabilities were compared to assess topological congruence among inference methods and between molecular markers.

## Results

### Phylogenetic analysis and taxonomic significance

**Figure 1.**
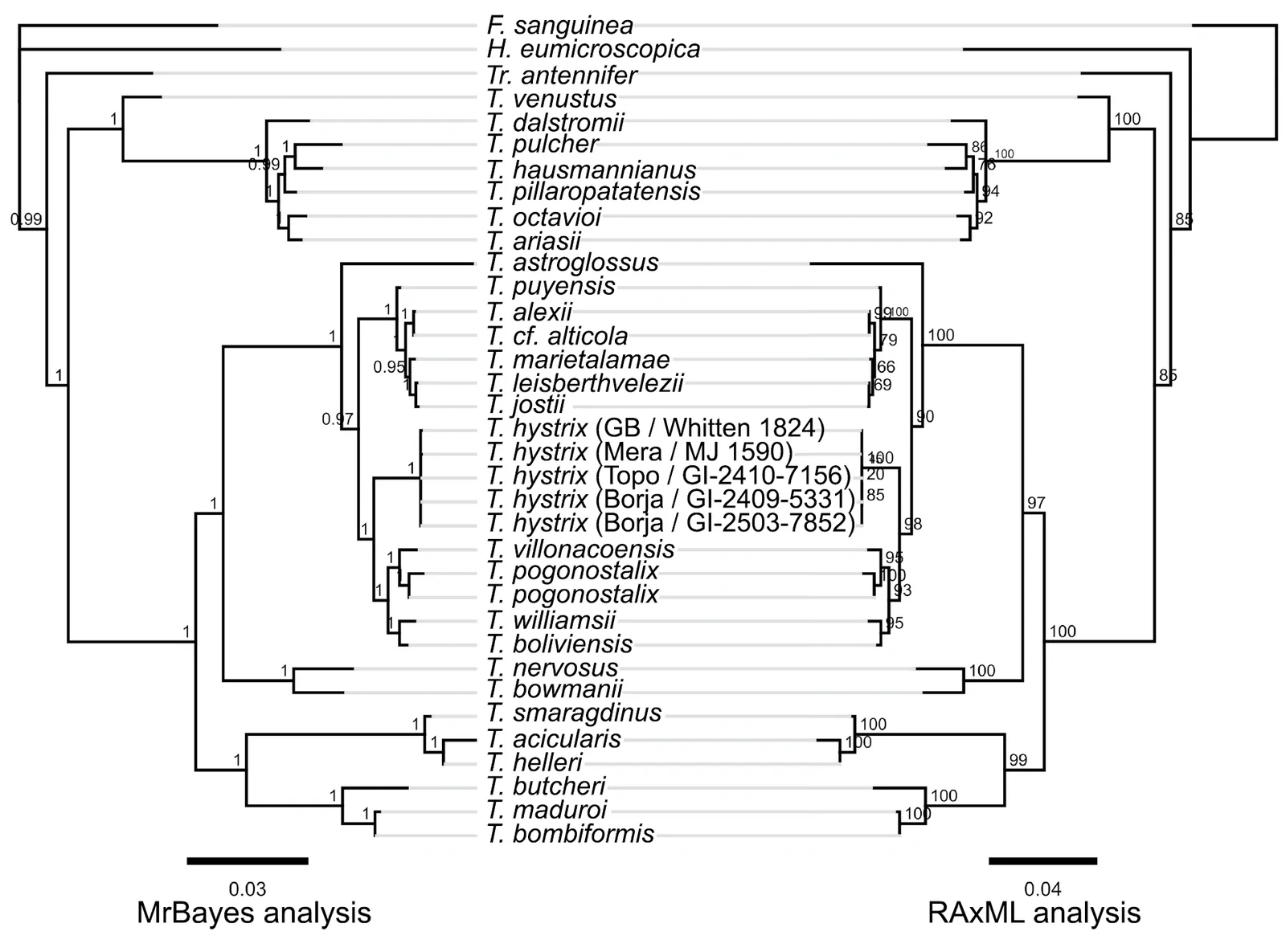
Phylogenetic trees inferred from the concatenated nrITS and *matK* markers. The tree on the left represents the 50% majority-rule consensus tree obtained from the Bayesian inference (BI) analysis in MrBayes (numbers at nodes represent posterior probabilities). The tree on the right represents the best-scoring maximum likelihood (ML) tree inferred with RAxML (numbers at nodes represent bootstrap support values (%) from 1,000 rapid bootstrap replicates). Scale bars represent the mean number of nucleotide substitutions per site. For *Telipogon hystrix*, the locality and field number are shown in parentheses; GB denotes GenBank.

Phylogenetic trees inferred from the nrITS marker recovered topologies that were highly consistent with those obtained from the plastid *matK* dataset (Suppl. materials 1, 2) and with the concatenated analysis. Therefore, only the phylogenetic tree derived from the concatenated dataset is presented and discussed herein, as it provides higher resolution (Fig. 1). Both Bayesian inference (BI) and maximum likelihood (ML) analyses yielded largely congruent topologies, recovering *Telipogon* as a monophyletic group with strong support (PP = 1.0; BS = 85). Within the genus, several internal clades were well resolved, with high bootstrap and posterior probability values, including lineages containing the newly described species *T. marietalamae* and *T. puyensis*. Both species were recovered within a clade (PP = 1.0; BS = 100) also including *T. alexii*, *T. cf. alticola*, *T. jostii*, and *T. leisberthvelezii*, all of which are miniature *Telipogon* species characterized by an unlobed column.

*Telipogon marietalamae* was recovered within a moderately supported subclade that also includes *T. jostii* and *T. leisberthvelezii* (PP = 0.95; BS = 66). Within this clade, *T. marietalamae* was resolved as the sister taxon to a lineage comprising *T. jostii* and *T. leisberthvelezii* (PP = 1.0; BS = 69). Although support for the internal relationships within this clade is inconsistent between inference methods, the placement of *T. marietalamae* as a distinct lineage was consistent across analyses.

*Telipogon puyensis* was recovered as sister to the lineage composed of *T. alexii*, *T. cf. alticola*, *T. jostii*, *T. leisberthvelezii*, and *T. marietalamae*, with strong support (PP = 1.0; BS = 100). This topology shows an early-branching position of *T. puyensis* among the Andean miniature *Telipogon* species with an unlobed column.

Accessions corresponding to *Telipogon hystrix* from Mera and Topo (Pastaza and Tungurahua Provinces, respectively), as well as a sequence from GenBank, together with accessions identified as *T. reventadorensis* from Borja (Napo Province), were all recovered within a single, strongly supported clade (PP = 1.0; BS = 100; Fig. 1). No consistent genetic differentiation or substructure was detected among these samples.

Overall, the phylogenetic results consistently recover *T. marietalamae* and *T. puyensis* as genetically distinct lineages, clearly separated from each other and from their closest relatives based on both nuclear and plastid markers. Therefore, the molecular evidence supports recognizing both taxa as distinct species.

In contrast, the lack of molecular differentiation between accessions attributed to *T. hystrix* and *T. reventadorensis* indicates that they represent a single evolutionary lineage and supports their treatment as a single species. This is supported by morphological information, which is discussed below.

### Taxonomic treatment

#### Telipogon marietalamae

Taxon classificationPlantaeAsparagalesOrchidaceae

N.Lapo-Gonzalez, M.M.Jiménez & Iturralde sp. nov.

38A99B2A-0607-57F5-A76D-733A00026667

urn:lsid:ipni.org:names:77422911-1

Fig. 2

##### Type.

Ecuador • Zamora Chinchipe, El Pangui: Pachicutza, near Santa Rita, 1,170 m, 22 January 2024, collected by Nadia Lapo, plant flowered in cultivation on 22 December 2025, coordinates omitted for conservation reasons, detailed locality data on the holotype, *M.M. Jiménez 2708* (Holotype HUTPL: 15849!).

**Figure 2.**
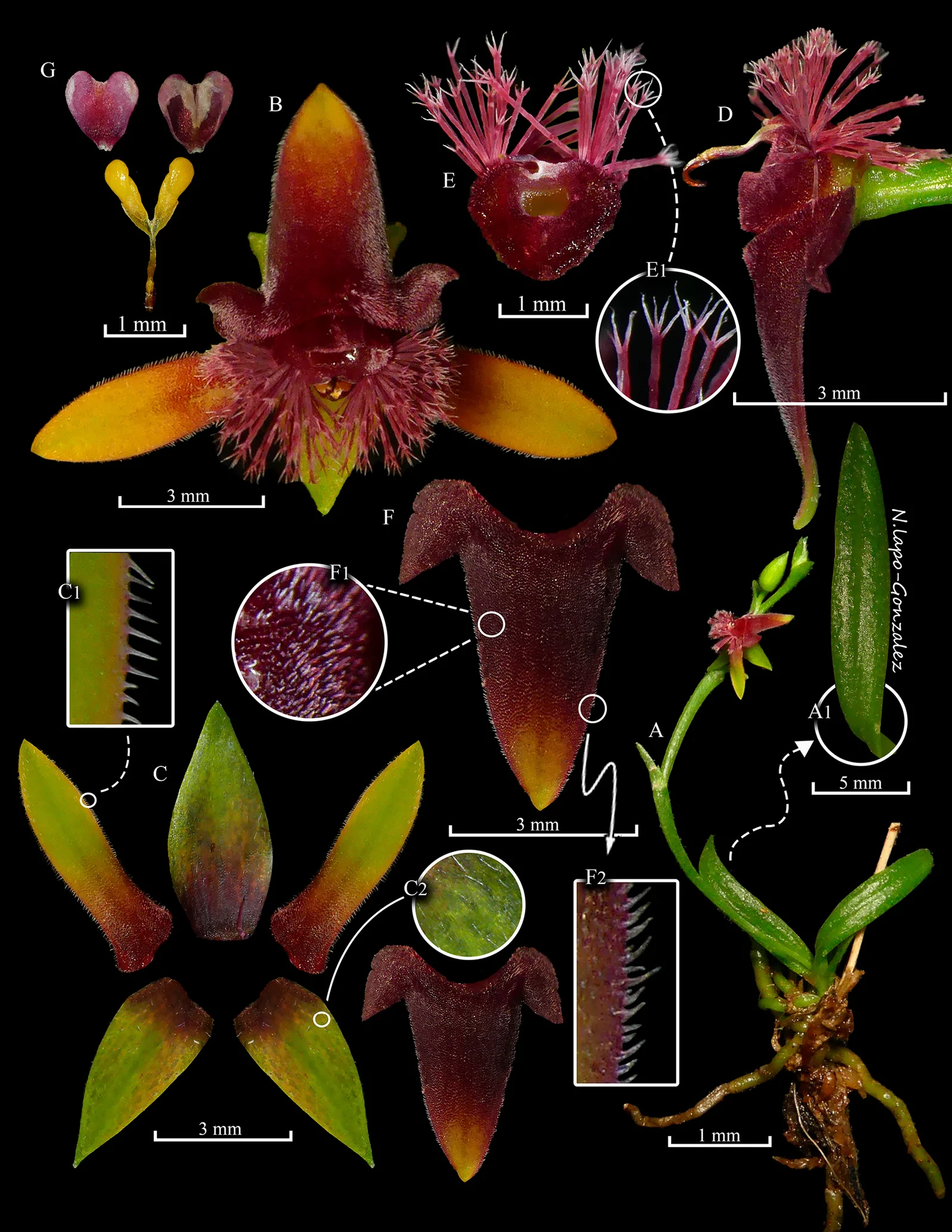
Lankester Composite Dissection Plate (LCDP) of *Telipogon marietalamae*. **A**. Habit with a close-up of the leaf (**A1**); **B**. Flower in frontal view; **C**. Dissected perianth with close-ups of the margin (**C1**) and indumentum of the sepals (**C2**); **D**. Column, lip, and ovary in lateral view; **E**. Column in frontal view with a close-up of the setae tips (**E1**); **F**. Lip in adaxial view with close-ups of the trichomes (**F1**) and margin (**F2**); **G**. Anther cap in dorsal view (left), ventral view (right), and pollinarium. Plate prepared by Nadia Lapo-González based on photographs by Marco M. Jiménez from the type specimen (**A, C–G**) and specimen *M.M. Jiménez 2625* (**B**).

##### Diagnosis.

The new species is similar to *Telipogon leisberthvelezii* by having yellowish sepals and petals, a reddish lip with a yellow apex, evident ovate-triangular lateral lobes, and two lateral tufts of stellate setae on the column. It differs in having 1-veined, ligulate-lanceolate petals (vs. 3-veined, narrowly obovate), a lip with a swollen base including the lateral lobes (vs. flat), and a narrower, ca. 2.5 mm wide, velvety mid-lobe of the lip (vs. 3.5 mm wide, minutely pilose), as well as denser tufts of setae, with 29–35 setae per tuft bearing 2–4 apical tips (vs. 16–20 setae per tuft, with 5–6 apical tips).

##### Description.

***Plant*** epiphytic up to 6 cm tall, including the inflorescence, caespitose, each new shoot produced appressed to and above the previous one; the old shoot withering as the new one matures; ***roots*** basal, white, flexuous, 1.1–1.7 mm in diameter, fleshy. ***Stem*** inconspicuous, ca. 2.0 mm long. ***Leaves*** distichous, articulated, 1.9–2.1 cm long, sub-coriaceous, thick; ***blades*** 2–4 per stem, linear-lanceolate, falcate, 18–19 × 3.0 mm, canaliculate, papillose-sub-verrucose adaxially, margin slightly revolute, apex subacute, inner leaf articulated to the stem, outer leaves articulated to conduplicate sheaths surrounding the stem. ***Inflorescences*** one per stem, racemose, indeterminate, erect to suberect, green, up to 5.5 cm long, up to 2–3 flowers produced singly, and spirally arranged; ***peduncle*** sub-ancipitous from the distal bract, ca. 26 mm long, gradually becoming narrow and terete towards the base; ***rachis*** 29 mm long, each segment somewhat narrowed towards the base and slightly compressed beneath each floral bract, subverrucose, ***floral bracts*** decurrent, green, conduplicate, triangular-ovate, 2.6–3.5 × 1.4–1.8 mm, sparsely pustulose, acute. ***Ovary*** emerald green, subterete, 1.7–1.9 × 0.8–1.0 mm, grooved; ***pedicel*** emerald green, falcate to slightly sigmoid, 3.5–3.7 mm long, somewhat compressed and grooved, sub-verrucose. ***Flowers*** 6.8–8.4 × 8.7–11.4 mm, non-resupinate. Sepals and petals stained with brown or dark mahogany at the basal fourth; sepals opaque lemon-green, petals matte yellow-orange with the darker margins; lip mahogany to dark vinaceous, mid-lobe yellow-orange at the apex; trichomes pale pink. ***Sepals*** reflexed, free, lanceolate, membranous, shallowly concave, very sparsely pubescent, pustulose adaxially, verrucose abaxially, margins entire, slightly involute; ***dorsal sepal*** 3-veined (lateral veins just reaching the middle), ca. 4.9 × 2.2 mm, apex somewhat attenuate; ***lateral sepals*** 1-veined, oblique, 4.5–4.7 × 2.0 mm, apex attenuate. ***Petals*** 1-veined, slightly falcate, ligulate-lanceolate, 5.6–6.0 × 1.5–1.9 mm, puberulous, slightly channeled along the midvein, base truncate, slightly thickened and widened, margins ciliate, retrorse, cilia getting shorter towards the apex, apex subacute, veins faint in coloration; base with two projecting, subacute auricles, the one facing the anther 0.3 mm long, the one facing the ovary 0.5 mm long. ***Lip*** 3-lobed, perpendicular to and surrounding the base of the column, sub-hastate, abaxial surface papillose, adaxial surface velvety, swollen at the base, including the lateral lobes, base subtruncate, margins flat, retrorsely ciliate, hairs white; ***mid-lobe*** 7-veined seen from material preserved in alcohol (lateral veins not reaching the apex), sub-triangular, 3.5–4.3 × 2.5 mm, slightly elevated, striate, apex subacute, incurved, shallow, veins faint; ***lateral lobes*** spreading, ovate-triangular, 1.9 × 0.9–1.2 mm, convex, apex acute and sometimes falcate. ***Column*** stout, dark vinaceous with paler trichomes, widely sub-cordiform, 1.8–2.0 × 1.9–2.1 mm, velvety; lateral margins dilated, with two tufts of setae arising from behind each edge, ventral edge rounded, protruding and swollen, forming an ovate, obtuse bump; ***setae*** 29–35 per tuft, fasciculate, up to 2.2 mm long, stellate with 2–4 white apical tips; ***clinandrium*** not hooded, superior margin emarginate, with a subcircular, elevated bump over it; ***stigma*** apical, transversely oblong, margins of the stigma slightly swollen; rostellum erect. ***Anther cap*** bilocular, dorsal, purplish brown, cordiform, 1.1 × 1.0 mm. ***Pollinarium*** ca. 2.0 mm long, with two pairs of unequal, narrowly obovoid, complanate pollinia, ***stipe*** ca. 1.0 mm long, viscidium uncinate. ***Fruits*** not seen.

##### Etymology.

The specific epithet honors Marieta de Jesús Alama, the paternal grandmother of the coauthor Nadia Lapo and the mother of Lester Lapo, a prominent orchid grower who discovered this species.

##### Phenology.

Flowering has been recorded *in situ* from January to November and *ex situ* during August, November, and December. Given this broad flowering period, no clear phenological pattern can be established.

##### Distribution, habitat, and ecology.

**Figure 3.**
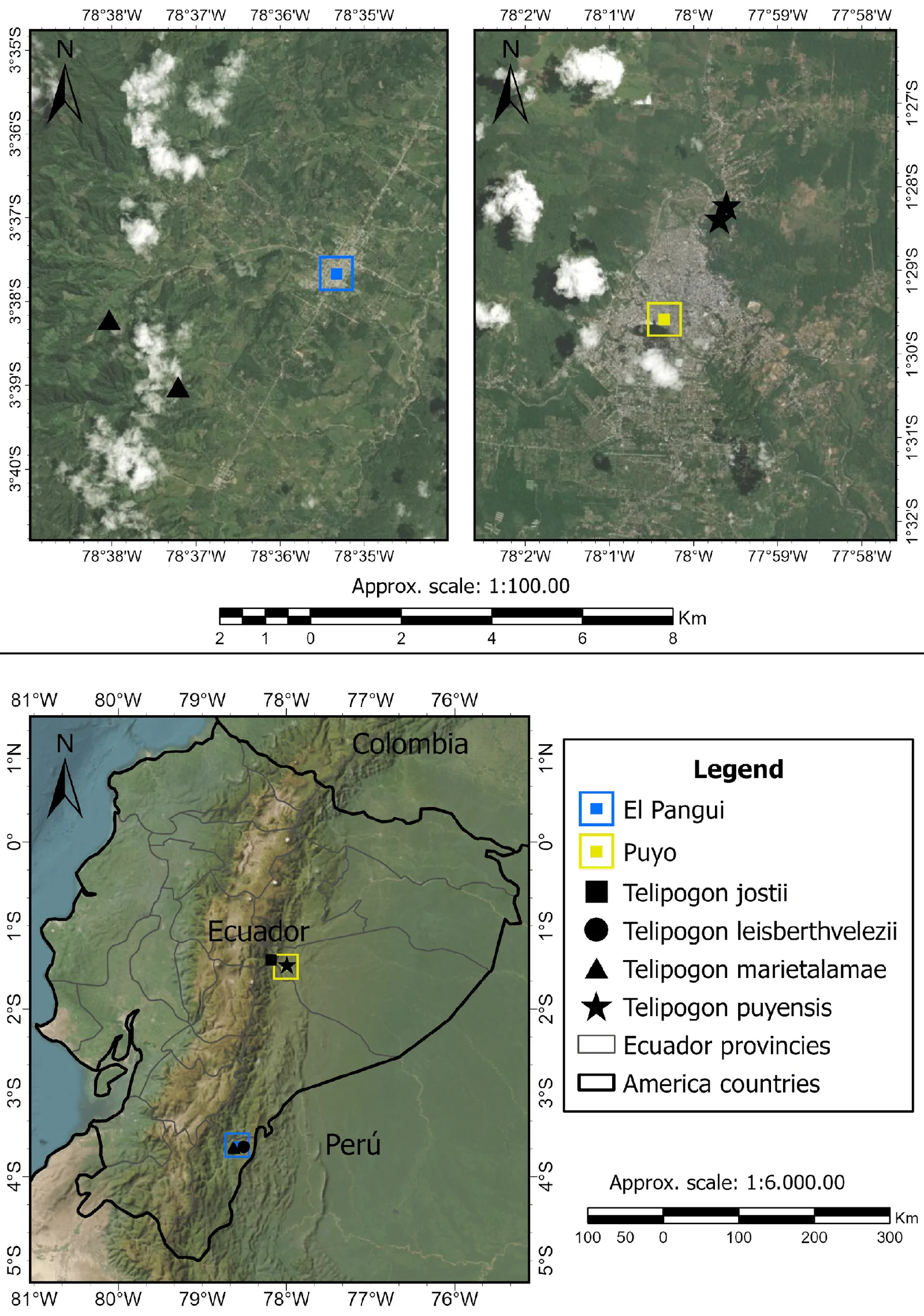
Distribution map of *Telipogon jostii*, *T. leisberthvelezii*, *T. marietalamae*, and *T. puyensis*. Produced by Henry X. Garzón-Suárez.

**Figure 4.**
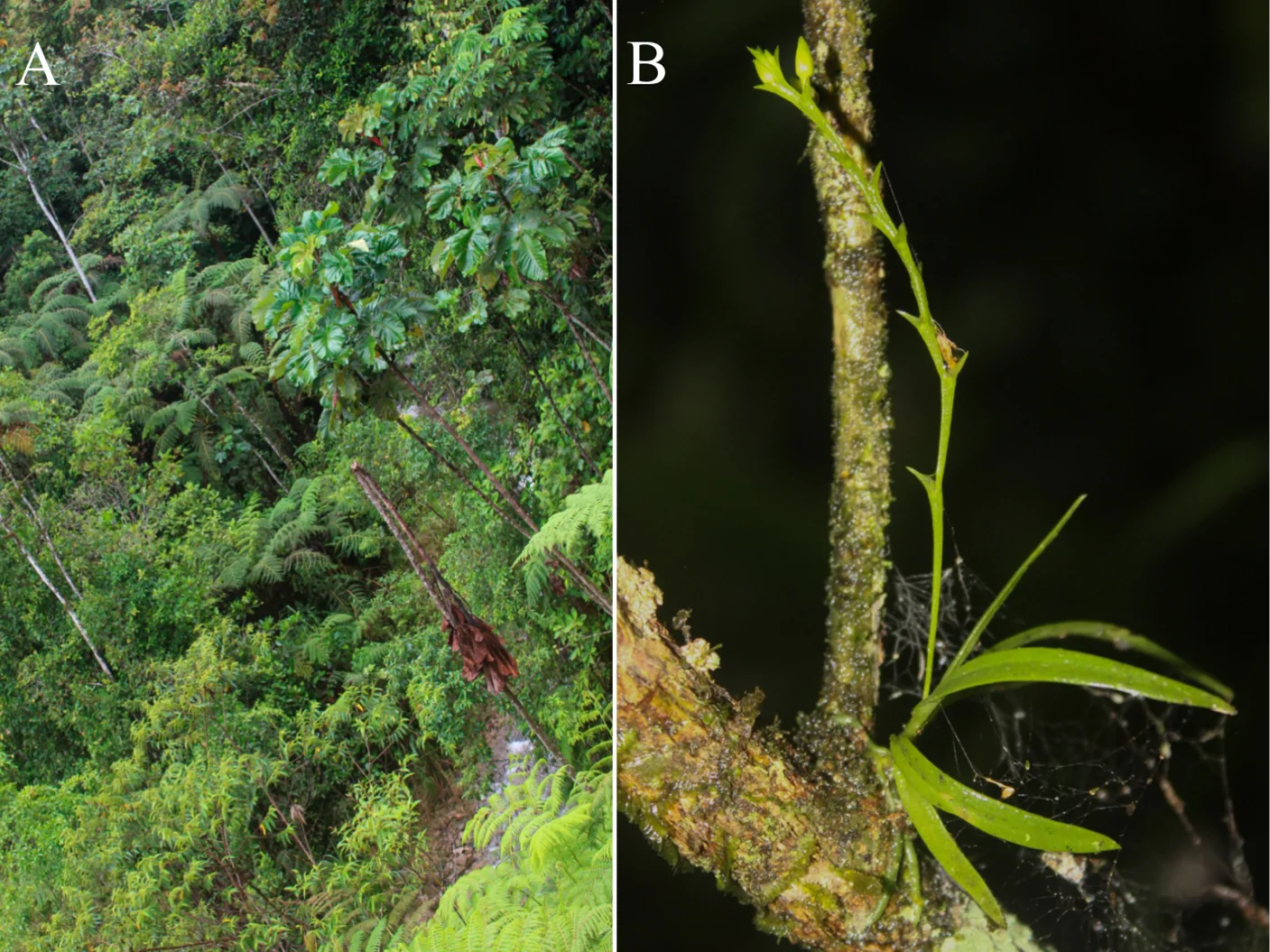
Habitat and holotype specimen of *Telipogon marietalamae*. **A**. Piedmont forest at the type locality; **B**. A mature, flowering *Telipogon marietalamae* growing on a *Palicourea* sp. Photographs by N. Lapo-Gonzalez.

Based on available records, *Telipogon marietalamae* occurs in secondary forests within the Andean foothills of Zamora Chinchipe Province, near El Pangui and Pachicutza (Figs 3, 4). The species is currently known from two localities. The type locality is in the Santa Rita neighborhood at an elevation of 1,170 m, where at least six individuals were observed. The second locality lies in the Simón Bolívar neighborhood at 1,317 m, where two individuals were observed (Fig. 3).

The ecosystem corresponds to the Evergreen piedmont forest of the Cordillera del Cóndor-Kutukú, code BsPa02 (Ministerio del Ambiente 2013; MAE 2026). Forests at the type locality are characterized by a canopy reaching up to 15 m in height and are dominated by species of *Cecropia* Loefl., *Neea* Ruiz & Pav., *Miconia* Ruiz & Pav., and *Palicourea* Aubl. Plants of *T. marietalamae* grow epiphytically on twigs and trunks covered with mosses and lichens in the understory, a few meters above the ground. On some phorophytes, individuals were observed growing in small clusters of one or two plants per host (Fig. 4). The new species occurs sympatrically with *Rodriguezia lanceolata* Ruiz & Pav. and *Telipogon astroglossus* Rchb.f.

##### Preliminary IUCN conservation assessment.

At present, *T. marietalamae* is known only from two nearby localities separated by approximately 2.3 km, each hosting a small population composed of a few individuals. Both localities are situated within the same catchment of the Zamora River, close to the urban centers of Pachicutza and El Pangui.

Based on current data, the area of occupancy (AOO) is estimated at 8 km^2^. The species is known from only two closely spaced localities, representing a single location *sensu*IUCN (2024). Due to the limited number of records, it is not possible to calculate the extent of occurrence (EOO); therefore, in accordance with IUCN guidelines, the EOO was inferred to be equal to the AOO. The habitat faces serious threats due to uncontrolled deforestation associated with multiple land-use pressures, including infrastructure development, dry-pit extraction of construction aggregates, and slash-and-burn clearing to establish cattle pastures and cacao and plantain plantations. Such activities cause ongoing degradation and loss within the valley.

Although the Andean foothills of Zamora Chinchipe Province remain poorly explored and additional subpopulations may occur, the currently available data indicate an extremely restricted geographic range, with AOO < 10 km^2^ and EOO < 100 km^2^, in a single location with an ongoing decline in habitat quality. Following the precautionary principle and considering the ongoing threats, classification of *T. marietalamae* as Critically Endangered (CR) under criteria B1ab(iii)+2ab(iii) is recommended based on IUCN (2024).

##### Additional material examined.

Ecuador • Zamora Chinchipe, El Pangui; Barrio Simón Bolivar, collected by Nadia Lapo, 1,317 m, 14 January 2026, plant flowered in cultivation on 18 March 2026, *M.M. Jiménez 2792* (paratype HUTPL!).

##### Taxonomic discussion.

*Telipogon marietalamae* and *T. leisberthvelezii* are similar in having indeterminate inflorescences bearing single flowers, yellowish petals provided with basal auricles, a reddish 3-lobed lip, and a column with two lateral tufts of long stellate setae. A detailed description of *T. leisberthvelezii* was provided by Jiménez et al. (2024). However, the two species differ in several floral characters, particularly in the morphology of the sepals, petals, lip, and column.

Sepals of *Telipogon marietalamae* differ in being opaque, lanceolate, sparsely pubescent, and shallowly concave (vs. translucent, ovate, glabrous, and concave). Petals are 1-veined and ligulate-lanceolate (vs. 3-veined, narrowly obovate). Petal auricles are straight, triangular, and subacute, reaching 0.3–0.5 mm long (vs. falcate, oblong, truncate, and up to 0.8 mm long).

**Figure 5.**
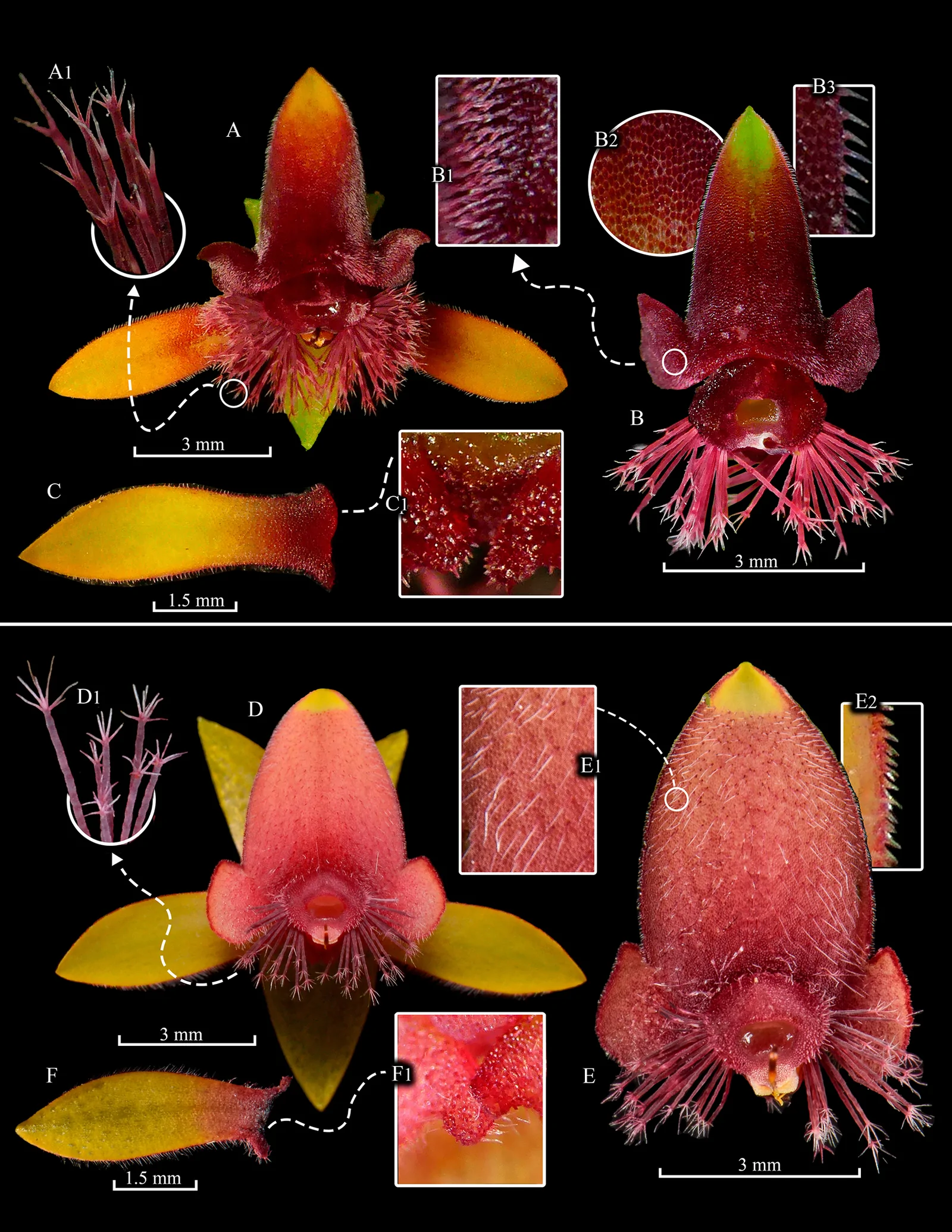
Floral comparison between *Telipogon marietalamae* and *T. leisberthvelezii*. **A–C**. *Telipogon marietalamae*; **A**. Flower, frontal view; **A1**. Close-up of the setae; **B**. Column and lip, frontal view; **B1**. Close-up of the lip lobes; **B2**. Close-up of the lip apex; **B3**. Close-up of the lip margin; **C**. Petal, frontal view; **C1**. Close-up of the petal lobes; **D–F**. *Telipogon leisberthvelezii*; **D**. Flower, frontal view; **D1**. Close-up of the setae; **E**. Column and lip, frontal view; **E1**. Close-up of the adaxial surface of the lip; **E2**. Close-up of the lip margin; **F**. Petal, frontal view; **F1**. Close-up of the petal lobes. Plate prepared by Nadia Lapo-Gonzalez based on photographs of the type specimens of *Telipogon marietalamae* by Marco M. Jiménez and *T. leisberthvelezii* by Henry X. Garzón-Suárez (**D–F**) and Marco M. Jiménez (**F1**).

The lip has a swollen base, including the lateral lobes, and a velvety adaxial surface (vs. flat and pilose). The mid-lobe is narrower, ca. 2.5 mm wide (vs. 3.5 mm wide). The lateral lobes are ovate-triangular (vs. widely subovate). The column is widely subcordiform and velvety (vs. subterete and papillose). The lateral tufts of setae are denser and longer, with 29–35 setae per tuft and reaching up to 2.2 mm long (vs. 16–20 setae per tuft reaching up to 1.5 mm long). The stigma is transversely oblong (vs. widely elliptic), with the basal margin bearing an obtuse, ovate bump centrally (vs. slightly protruding) (Table 2; Fig. 5).

**Table 2.** Summary of principal differences between morphologically similar species.

**Character**	***T. marietalamae***	***T. leisberthvelezii***	***T. puyensis***	***T. jostii***
**Inflorescence**	Subancipitous, more compressed beneath each floral bract; unbranched	Subancipitous, more compressed beneath each floral bract; unbranched	Terete; sometimes 1–3-branched	Subancipitous, more compressed beneath each floral bract; unbranched
**Flower color**	Yellow-orange petals; mahogany to dark vinaceous lip with yellow apex; dark mahogany column and setae	Yellow petals; dark pink lip with yellow apex; purple column and setae	Burgundy petals; maroon lip; white-pink column; burgundy setae	Vinaceous-red petals and lip; dark purple column and setae
**Petals**	Ligulate-lanceolate, spreading, 1-veined, puberulous with retrorse-ciliate margins, 5.6–6.0 × 1.5–1.9 mm	Narrowly obovate, spreading, 3-veined, ciliate-pilose, becoming pubescent and then glabrous toward the apex, with retrorse-ciliate margins, 5.1–5.5 × 1.6–1.9 mm	Oblanceolate, arcuate, incurved, 1-veined, glabrous, 4.5–4.7 mm long	Elliptic, spreading, 1-veined, glabrous, 3 × 1.7 mm
**Lip**	Spreading, with swollen base, including the lateral lobes	Spreading, with flat base and lobes	Reflexed downward, with swollen base, including the lateral lobes	Spreading, with swollen base, including the lateral lobes
**Lip mid-lobe**	Subtriangular, 3.5–4.3 × 2.5 mm	Subtriangular, 4.5 × 3.5 mm	Subtriangular, 3.1–3.3 × 2.1 mm	Oblong, 3.5–4.7 × 1.5–1.9 mm
**Lip lobes**	Ovate-triangular, sometimes with a falcate apex; spreading	Widely subovate; spreading	Subdolabriform; reflexed backward	Ovate-triangular, sometimes with a falcate apex; spreading
**Column**	Widely subcordiform, with dilated lateral margins	Subterete	Ancipitous, with dilated lateral margins	Subterete
**Column setae**	Acicular with stellate apex, up to 2.2 mm long; 29–35 setae per tuft bearing 2–4 long apical tips	Acicular with stellate apex, up to 1.5 mm long; 16–20 setae per tuft, with 5–6 long apical tips	Acicular, hispid from near the middle to the apex, up to 1.9 mm long; 20–30 setae per tuft	Acicular with stellate apex, up to 1.2 mm long; 35–45 setae per tuft, with 4–6 very short apical tips
**Stigma**	Transversely oblong	Widely elliptic	Narrowly reniform	Elliptic

**Figure 6.**
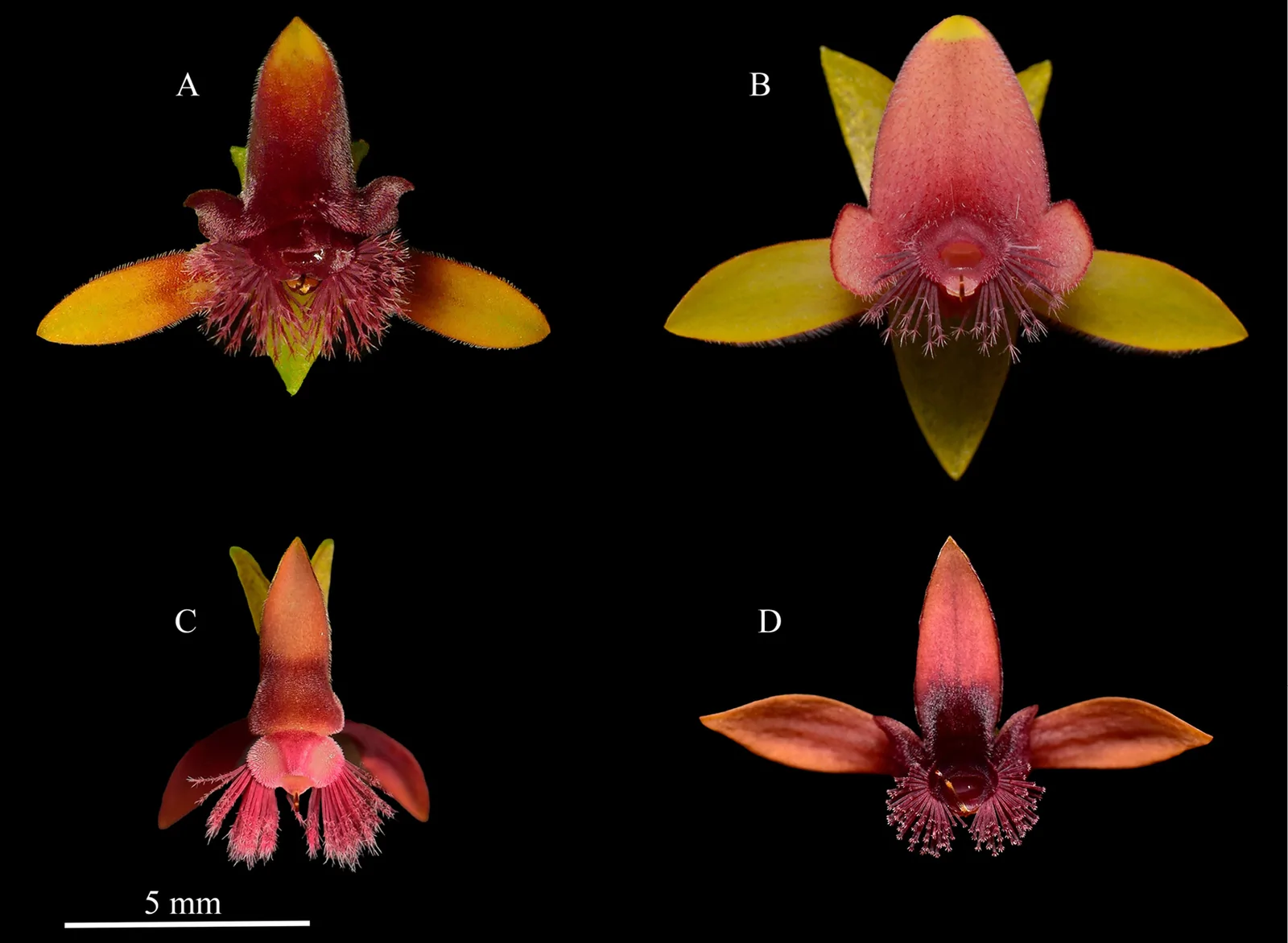
Flowers of *Telipogon* species. **A**. *T. marietalamae*; **B**. *T. leisberthvelezii*; **C**. *T. puyensis*; **D**. *T. jostii*.

The new species is also similar to *Telipogon jostii*, with both species sharing distichous leaves, oblique lateral sepals, and a 3-lobed lip. However, they differ in several floral characters: flowers of *T. marietalamae* have yellow-orange petals and a mahogany to dark vinaceous lip with a yellow apex (vs. vinaceous-red petals and lip). The sepals are larger: the dorsal sepal is 4.9 × 2.2 mm, with a somewhat attenuate apex (vs. 3.0 × 1.5 mm and blunt), and the lateral sepals are 4.5–4.7 × 2.0 mm (vs. 3 × 1.5 mm). Petals are ligulate-lanceolate and larger, 5.6–6.0 × 1.5–1.9 mm (vs. 3 × 1.7 mm and elliptic). The column is widely subcordiform (vs. subterete), with tufts of 29–35 setae up to 2.2 mm long (vs. tufts of 35–45 setae up to 1.2 mm long) (Table 2; Fig. 6).

There are also differences in ecology and distribution among these species. *Telipogon marietalamae* occurs in the foothills of the eastern slope of the Andes, while *T. leisberthvelezii* is restricted to the western slopes of the Cordillera del Cóndor, an isolated mountain range situated east of the main Andean chain. Both species inhabit mature secondary forests in Zamora Chinchipe Province, southeastern Ecuador. The new species grows predominantly in the forest understory, whereas *T. leisberthvelezii* is usually found in the canopy. In contrast, *T. jostii* is restricted to the provinces of Tungurahua and Pastaza in central-eastern Ecuador and has been found growing on twigs of guava trees. All three species occur at relatively similar elevations, ranging from approximately 1100 to 1350 m (Fig. 3).

#### Telipogon puyensis

Taxon classificationPlantaeAsparagalesOrchidaceae

M.M.Jiménez, D.Gut. del Pozo & Iturralde sp. nov.

715CF5FA-44AB-5E97-846F-C17F128C13DF

urn:lsid:ipni.org:names:77422912-1

Fig. 7

##### Type.

Ecuador • Pastaza, Puyo, sendero ecoturístico del río Puyo; 941 m (coordinates omitted for conservation reasons; detailed locality data available on the herbarium type specimens), 26 October 2024, plant flowered in cultivation on 25 November 2025, *M.M. Jiménez, G.A. Iturralde & D. Gutiérrez del Pozo 2438* (Holotype: HUTPL 15798!).

**Figure 7.**
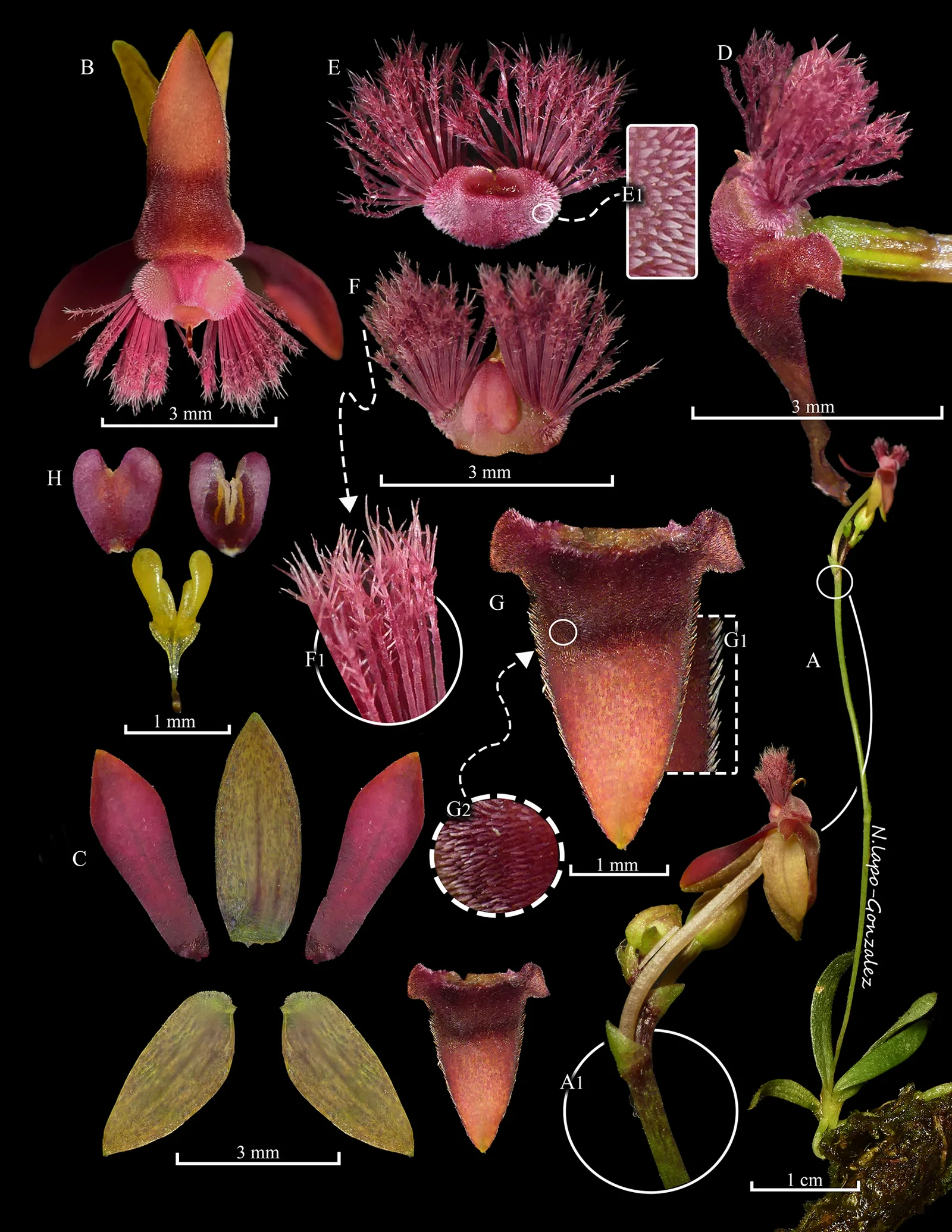
Lankester Composite Dissection Plate (LCDP) of *Telipogon puyensis*. **A**. Habit with a close-up of the rachis (**A1**); **B**. Flower in frontal view; **C**. Dissected perianth; **D**. Column, lip, and ovary in lateral view; **E**. Column in ventral view with a close-up of the indumentum (**E1**); **F**. Column in dorsal view with a close-up of the setae tips (**F1**); **G**. Lip in adaxial view with close-ups of the margin (**G1**) and trichomes (**G2**); **H**. Anther cap in dorsal view (left), ventral view (right), and pollinarium. Plate prepared by Nadia Lapo-González based on photographs by Marco M. Jiménez (**A1, C–H**) and G. Iturralde (**A, B**) from the type specimen.

##### Diagnosis.

*Telipogon puyensis* is similar to *T. jostii* in its dark red-purplish flowers, petals with entire margins, the 3-lobed lip with a dominant mid-lobe and two smaller, recurved lateral lobes, and the column bearing two dense lateral tufts of apically bristled setae. It differs by its terete, sometimes 1–3 branched inflorescence (vs. sub-ancipitous more compressed beneath each floral bract, unbranching), burgundy-maroon flowers with a white and pink column (vs. vinaceous-red with dark purple column), incurved, arcuate, oblanceolate petals, 4.5–4.7 mm long (vs. elliptic, erect, 3 mm long), a lip with a sub-triangular mid-lobe (vs. oblong), and a column bearing setae which are hispid from near the middle to the apex (vs. apically stellate setae).

##### Description.

***Plant*** epiphytic up to 14 cm tall, including the inflorescence, caespitose, each new shoot produced appressed to and above the previous one; the old shoot withering as the new one matures; ***roots*** basal, white, flexuous, 0.8–1.0 mm in diameter, fleshy. ***Stem*** inconspicuous, ca. 2.5 × 0.8 mm. ***Leaves*** distichous, articulated, 9.0–22 mm long, sub-coriaceous; ***blades*** 3–4 per stem, ligulate to oblong, apex subacute, 14–19 × 2 mm, flat, canaliculate, papillose-sub-verrucose adaxially, inner leaf articulated to the stem, outer leaves articulated to conduplicate sheaths surrounding the stem. ***Inflorescences*** one per stem, lateral, erect to suberect, racemose, indeterminate, sometimes 1–3 branching from the distal peduncle bract, green to heavily splashed with purple-brown, up to 12.5 cm long, up to 6–7 flowers produced singly and spirally arranged; ***peduncle*** terete, 5.2–10.9 cm long, ca. 0.1 cm in diameter; ***rachis*** terete, 6.0–12.2 × 1.0 mm, pustulose; ***floral bracts*** decurrent, green translucent sometimes suffused with brown-purple at the base, conduplicate, triangular-ovate, 1.3–1.9 × 1.2–1.3 mm, sparsely pustulose. ***Ovary*** light green to green, stained with brown at the ribs, subterete, 2.0–4.4 × 0.9 mm, grooved; ***pedicel*** pale green to lavender, falcate, slightly sigmoid, 8.7–9.0 mm long, somewhat compressed and grooved, sub-verrucose. ***Flowers*** 5.7–7.6 × 5.6–8.1 mm, non-resupinate. Sepals and petals matte, papillose; sepals pale yellowish-brown, barred with faint brownish purple, becoming intense to the veins and the base, petals burgundy, lip with the lobes and the mid-lobe maroon, light brownish pink above the middle, trichomes pink. ***Sepals*** 3-veined, reflexed, free, narrowly elliptic, membranous, concave, sparsely verrucose abaxially, pustulose adaxially, margins entire, slightly involute, apex shortly attenuate; ***dorsal sepal*** 4.1–4.3 × 1.3–1.6 mm (midvein reaching the apical third, lateral veins reaching the middle third); ***lateral sepals*** oblique, 3.6–4.3 × 1.4–1.6 mm. ***Petals*** 1-veined, incurved, arcuate, oblanceolate, 4.5–4.7 × 1.4–1.5 mm, reflexed, glabrous, base truncate, slightly thickened, margins entire, apex slightly concave, subacute, vein inconspicuous. ***Lip*** 3-lobed, reflexed ventrally, surrounding the base of the column, sub-sagittate, abaxial surface papillose, sparsely papulose, adaxial surface velvety and swollen somewhat above the middle, including the lateral lobes, base truncate, margins antrorse-pectinate, hairs white; ***mid-lobe*** 9-veined seen from material preserved in alcohol, sub-triangular when expanded, 3.1–3.3 × 2.1 mm, convex above the middle, papillose in the upper half, retrorse at the margins, apex subacute, with a longitudinal shallow sulcus ending in the middle third of the blade, veins obscure; ***lateral lobes*** recurved, sub-dolabriform, 1.0 × 0.4–0.6 mm, convex, apex subacute, reflexed embracing the column. ***Column*** sessile, white-pink with white, short, thick trichomes, ancipitous, 1.5–1.7 × 2.0 mm, papillose-echinate, lateral margins dilated and rounded, each bearing one tuft of setae behind the margin, ventral margin darker, with a longitudinal ridge in the middle reaching the stigma; ***setae*** 20–30 per tuft, burgundy, up to 1.9 mm long, hispid from near the middle to the apex, with paler tips; ***clinandrium*** not hooded, emarginate in the superior margin; ***stigma*** apical, narrowly reniform, margins of the stigma slightly swollen; ***rostellum*** erect. ***Anther*** dorsal, anther cap bilocular, light burgundy, turning brown to the base, narrowly cordiform, 1.0 × 0.7 mm. ***Pollinarium*** 1.6 mm long, with two pairs of unequal, narrowly obovoid, complanate pollinia, ***stipe*** ca. 0.9 mm long, ***viscidium*** uncinate. ***Fruits*** not seen.

##### Etymology.

The specific epithet refers to Puyo, the name of both the town, which is the capital of Pastaza Province, and the river where the new species was discovered. Puyu or Puyo means fog or cloud in the pre-Columbian Quichua language.

##### Phenology.

Flowering has been recorded from July to November.

##### Distribution, habitat, and ecology.

*Telipogon puyensis* is currently known only from the vicinity of the ecological trail along the Puyo River in Pastaza Province, eastern Ecuador (Fig. 3). The species occurs in a highly disturbed area strongly affected by urban expansion. Individuals were observed growing on remnant trees along the river margins and on ornamental non-native shrubs within landscaped private areas located very close to the Puyo River, at the western edge of the Amazon basin in Ecuador.

The area belongs to the ecosystem classified as Northern Andean Eastern Piedmont Evergreen Forest, code BsPn03, characterized by a humid to hyper-humid climate (Ministerio del Ambiente 2013; MAE 2026).

The riverside urban park, extending for approximately 3.5 km along the riverbank, still preserves large *Vochysia* Aubl. trees and *Dacryodes* Vahl trees, together with rare and endemic species such as *Magnolia kichuana* A.Vázquez, F.Arroyo & Á.J.Pérez and the recently described *Browneopsis puyensis* D.A.Neill & Asanza. Despite the high level of anthropogenic disturbance, the adjacent ecological trail retains structural and floristic elements of a remnant primary forest, as previously documented for the riverside urban park of Puyo (Neill and Asanza 2014).

The new species was found growing epiphytically on *Brunfelsia grandiflora* D.Don and *Syzygium jambos* (L.) Alston. It occurs sympatrically with other orchid species, including *Maxillaria reichenheimiana* Endrés & Rchb.f., *Pleurothallis sphaerantha* Luer, and *Sobralia rosea* Poepp. & Endl.

##### Preliminary IUCN conservation assessment.

*Telipogon puyensis* is currently known from two subpopulations separated by less than 500 m in the vicinity of the ecological trail in Puyo, Pastaza Province, Ecuador. Both subpopulations occur in a highly disturbed landscape affected by ongoing urban expansion, infrastructure development, and habitat fragmentation. The species has been recorded exclusively on remnant trees along river margins and on ornamental non-native shrubs within a modified environment. Further field surveys are needed to better assess population size, the existence of additional localities, and the long-term viability of the species in highly perturbed environments. Based on current data, the AOO is estimated at 4 km^2^. Due to the lack of additional known localities, the EOO was inferred to be equal to the AOO.

A recent study identified urban growth and land-use change as the principal threats to biodiversity conservation in the area, while the conservation of peri-urban natural ecosystems is not currently a priority within local urban planning frameworks (Huera-Lucero et al. 2020). These trends indicate a continuing decline in the extent and quality of habitat available for the species. Considering its extremely restricted EOO and AOO, occurrence at a single threat-defined location, and inferred continuing decline in habitat quality, *T. puyensis* is here assessed as Critically Endangered (CR) under criteria B1ab(iii)+2ab(iii), according to the IUCN Red List Categories and Criteria (IUCN 2024).

##### Additional material examined.

Ecuador • Pastaza, Puyo, sendero ecoturístico del río Puyo; 941 m (coordinates omitted for conservation reasons; detailed data on the herbarium specimen), 26 October 2024, *G.A. Iturralde GI-2410-7357* (Paratype: QCNE!).

##### Additional *Telipogon* species examined.

***Telipogon alexii***: Ecuador • Pichincha, Nanegalito, west of Quito to Nanegal, 1,400 m, June 1983, *Hirtz 992* (Holotype: SEL photo!). ***Telipogon alticola***: Ecuador • Napo, 1 km east of Cuyuja, km 73 road Quito to Baeza, 2,400 m, 5 December 1985, *Dodson 16265* (Holotype: MO photo!). ***Telipogon astroglossus***: Peru • Chachapoyas, *Matthews s.n*. (Holotype: G photo!). ***Telipogon embreei***: Ecuador • Zamora-Chinchipe, km 34 Loja-Zamora, 1,500 m, 11 August 1958, *Dodson & Frymire 402* (SEL photo!). ***Telipogon hystrix***: Ecuador • Tungurahua, near Río Negro on the road from Baños to Puyo, 1,500 m, 20 February 1963, *Dodson & Thien 2290* (Holotype: SEL photo!). ***Telipogon jostii***: Ecuador • Tungurahua, Topo, finca cercana al centro del pueblo, 1,320 m, 26 October 2024, *G. Iturralde GI-2410-7088* (QCNE!). ***Telipogon leisberthvelezii***: Ecuador • Zamora Chinchipe, Cordillera del Cóndor, 1,258 m, 27 September 2022, *L. Vélez-Abarca 74* (Holotype: ECUAMZ!). ***Telipogon morganiae***: Ecuador • Cotopaxi, near Macuchi, road Quevedo-Latacunga, 1,500 m, 26 July 1979, *Dodson, Morgan & Fallen 8557* (Holotype: SEL photo!). ***Telipogon nigropurpureus***: Colombia • Boyacá: Arcabuco, cañón del río Pómeca, 2,300 m, 9 July 2001, *C. Barrera sub P. Ortiz 1133* (holotype: HPUJ photo!). ***Telipogon pogonostalix***: Ecuador • *Spruce R. 6135* (Holotype: G photo!). ***Telipogon villonacoensis***: Ecuador • Loja, western cordillera of the Loja basin, near Loja; 2565 m; 21 February 2023, *M. Jiménez 1560* (Holotype: HUTPL!). ***Telipogon williamsii***: Colombia • around Pipulquor, western slope of the west Andes of Tuquerres, 1,500–1,700 m, July–August, *Lehmann 8356* (Holotype: AMES photo!).

##### Taxonomic notes.

*Telipogon puyensis* is distinguished from *T. jostii* by the characters outlined in the diagnosis (Table 2; Fig. 6). Additional differences are the burgundy petals, maroon lip, white-pink column, and burgundy setae (vs. vinaceous-red petals and lip and dark purple column and setae), as well as the incurved base of the lip, with the lateral lobes directed posteriorly (vs. extended lateral lobes). To the best of current knowledge, *T. puyensis* is the only species of miniature *Telipogon* in which the column setae are hispid from near the middle to the apex. Additionally, *T. puyensis* seems to be distributed at lower elevations, ca. 900 m, in the northeastern Andean piedmont forest (code BsPn03; MAE 2026), compared with *T. jostii*, which is distributed at ca. 1,300–1,400 m in the northeastern montane forests (code BsBn01; MAE 2026) (Fig. 3).

### Taxonomic notes on the synonymy of *Telipogon reventadorensis* with *T. hystrix*

**Figure 8.**
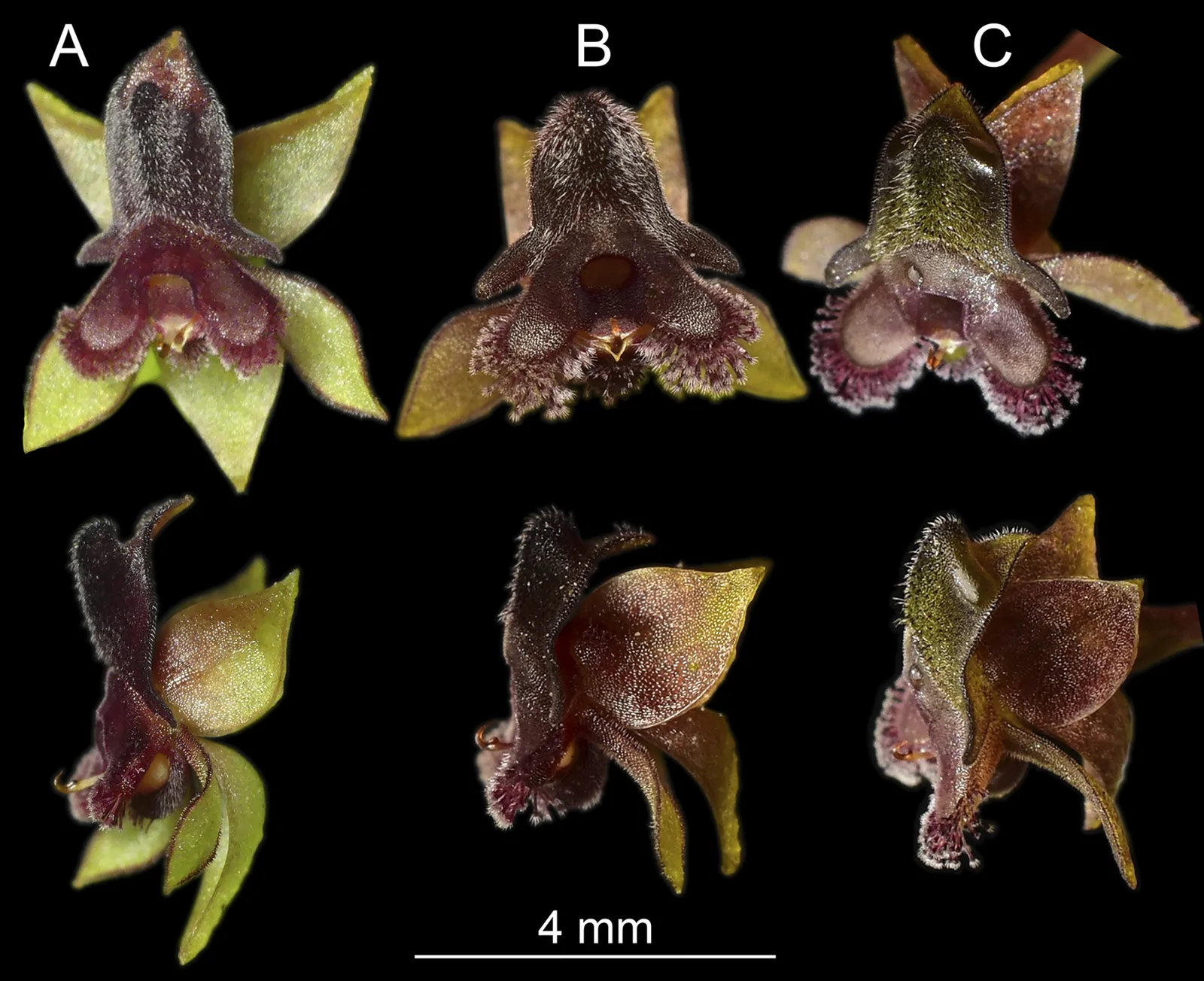
Flowers of *Telipogon hystrix* from different localities. **A**. Near Mera, Pastaza Province (*M.M. Jiménez MJ-1590*; photo voucher); **B**. Topo, Tungurahua Province (*G. Iturralde GI-2410-7156*; QCNE!); **C**. Near Borja, Napo Province (*G. Iturralde GI-2409-5331*; QCNE!). Photographs by Marco Jiménez (**A**) and Gabriel Iturralde (**B, C**).

*Telipogon hystrix* was originally described as *Stellilabium hystrix* by Dodson and Dodson (1982) based on plants collected from Tungurahua Province, central-eastern Ecuador. In contrast, *Telipogon reventadorensis* was described later by Dodson (2004) from the Volcán Reventador area in Napo Province, approximately 150 km north of the type locality of *T. hystrix*. In the protologue of *T. reventadorensis*, the species was compared with *T. boliviensis*, but no comparison was made with *T. hystrix*, despite their morphological similarity. The holotype of *Telipogon reventadorensis* (Hirtz 2127, ex RPSC) was unavailable for direct comparison with the type of *T. hystrix*. Although searches were undertaken in MO, SEL, QCNE, and QCA, where portions of the former RPSC collection are known to be housed, the specimen was not located. However, a detailed comparison of the original descriptions, illustrations (Dodson and Dodson 1982), a photograph (Dodson 2004), and newly collected living plants from multiple localities (Fig. 8) revealed no consistent diagnostic characters separating *T. reventadorensis* from *T. hystrix*. On the contrary, both taxa share a unique and concordant suite of morphological characters, including a subterminal inflorescence; obovate to spatulate petals with ciliate margins and acute apices; a lip with a sagittate base and a prominent, callus-like swelling in the lower half of the blade bearing retrorse, spine-like hairs; a flat, often reflexed lip apex; and a short column with two large lateral lobes whose apices are densely covered with stellate setae (Fig. 8).

The molecular phylogenetic analyses further support this morphological congruence. Accessions from near the original localities of *T. hystrix* and *T. reventadorensis* across Pastaza, Tungurahua, and Napo Provinces were recovered within a single, strongly supported clade (PP = 1.0; BS = 100; Fig. 1), with no supported genetic divergence among them based on nrITS and plastid *matK* markers. This lack of genetic distinctiveness is consistent with the observed morphological continuity across populations. Based on the absence of stable diagnostic morphological characters and consistent molecular evidence, *T. reventadorensis* is here regarded as conspecific with *T. hystrix* and is therefore reduced to synonymy. As a result, the known distribution of *T. hystrix* is extended beyond its previously documented range in Tungurahua at 1,500 m to include additional populations in Pastaza and Napo Provinces at elevations of 1,150–1,500 m.

#### Telipogon hystrix

Taxon classificationPlantaeAsparagalesOrchidaceae

(Dodson) N.H. Williams & Dressler, Lankesteriana 5(3): 170. 2005.

C8BDA632-A557-57B3-8344-EDCABCBE582B

Fig. 8

= Telipogon reventadorensis N.H.Williams & Dressler, Lankesteriana 5(3): 170. 2005. Type: ECUADOR • Napo, Baeza to Lago Agrio, volcán Reventador: Hirtz 2127 (holotype ex RPSC – unavailable for this study). syn. nov.
Stellilabium vulcanicum Dodson & Hirtz, C.H.Dodson, Native Ecuadorian Orchids 5: 1181 (2004).
Dipterostele hystrix (Dodson) Garay & G.A.Romero, Harvard Pap. Bot. 3: 57 (1998).
Stellilabium hystrix Dodson, Icon. Pl. Trop. 5: t. 496 (1982)

##### Type.

Ecuador • Tungurahua, near Río Negro on the road from Baños to Puyo, 1,500 m, 20 February 1963, *Dodson & Thien 2290* (holotype: SEL photo!).

##### Additional material examined.

(coordinates omitted for conservation reasons; detailed data on the herbarium specimen) Ecuador • Tungurahua, near Río Negro on the road from Baños to Puyo, 1,500 m, 20 February 1963, *Dodson & Thien 2290* (isotype: SEL!) • Tungurahua, Topo; 1,320 m, 25 October 2024, *G. Iturralde GI-2410-7156* (QCNE!) • Napo, Borja, vía a río Borja; 1,159 m, 30 October 2023, *G. Iturralde GI-2409-5331* (QCNE!) • Same locality, 30 October 2023, *G. Iturralde GI-2409-5357* (QCNE!) • Same locality, 30 March 2025, *GI-2503-7852* (QCNE!) • Pastaza, near Mera, 1,301 m, 25 November 2022, M.M Jiménez & D. Gutiérrez del Pozo MJ-1590 (photographic record Fig. 8).

## Discussion

A combination of morphological examination, phylogenetic analyses, and geographic data supports the recognition of *T. marietalamae* and *T. puyensis* as two new and distinct species within the genus, both belonging to the miniature *Telipogon* group. These two species were recovered as part of a clade of Andean miniature *Telipogon* species that share the absence of a lobed column.

Despite moderate support values for some internal relationships, particularly within the clade containing *T. marietalamae*, the placement of both new taxa as genetic entities distinct from other *Telipogon* species was consistent across inference methods and datasets. Furthermore, morphological comparisons between the most similar species provide strong support for recognizing both taxa as independent species.

The discovery of these two species reinforces the importance of the eastern Andean foothills as a center of diversification for miniature *Telipogon*.

Both new species appear to have highly restricted distributions and small population sizes, making them particularly vulnerable to habitat disturbance. The recognition of these narrowly endemic species highlights the urgent need for further botanical exploration and conservation assessment in these regions, which remain among the most biodiverse and increasingly threatened areas of Ecuador.

This study also contributes to a better understanding of species limits within miniature *Telipogon*. In particular, the combined molecular and morphological evidence indicates that *Telipogon reventadorensis* is not genetically or morphologically distinguishable from *T. hystrix*. Accessions attributed to both names form a single, strongly supported clade and fall within a shared range of morphological variation. This integrative evidence supports the synonymization of *T. reventadorensis* under *T. hystrix*, thereby improving taxonomic clarity and nomenclatural stability within the group.

### Key to Ecuadorian species of miniature *Telipogon*

**Table:** 

1	Leaves not articulated	**2**
–	Leaves articulated	**4**
2	Column not winged	***Telipogon sonia-juaniorum***
–	Column winged	**3**
3	Apex of the lip upturned, hooked	***Telipogon microglossus***
–	Apex of the lip flat	***Telipogon morganiae***
4	Inflorescence terete	**5**
–	Inflorescence sub-ancipitous to ancipitous	**9**
5	Column terete to ancipitous	**6**
–	Column transverse, bilobed	**7**
6	Petals shaggy adaxially, margins ciliolate; column setae stellate	***Telipogon nigropurpureus***
–	Petals glabrous; column setae hispid from near the middle to the apex	***Telipogon puyensis***
7	Column lobes and clinandrium with simple setae	***Telipogon astroglossus***
–	Column lobes with stellate setae and clinandrium with simple or stellate setae	**8**
8	Stellate setae on column lobes and simple setae on clinandrium	***Telipogon embreei***
–	Stellate setae on both the clinandrium and column lobes	***Telipogon hystrix* (= *T. reventadorensis* syn. nov.)**
9	Lip with inconspicuous basal auricles < 0.3 mm long	**10**
–	Lip with conspicuous basal lobes ≥ 0.6 mm long	**11**
10	Lip 3-veined	***Telipogon alexii***
–	Lip 5-veined	***Telipogon villonacoensis***
11	Labellar margin of the column extending over the base of the lip	***Telipogon williamsii***
–	Labellar margin of the column not extending	**12**
12	Flowers with petals and lip predominantly yellow-green	**13**
–	Flowers with yellow, yellow-orange or vinaceous petals and dark mahogany, vinaceous, pink or purple lip	**14**
13	Lateral lobes of the lip 0.6–0.7 mm long, falcate	***Telipogon alticola***
–	Lateral lobes of the lip 1–1.4 mm long, recurved	***Telipogon pogonostalix***
14	Mid-lobe of the lip wide; 3.5 mm wide, lateral lobes widely subovate	***Telipogon leisberthvelezii***
–	Mid-lobe of the lip narrow; up to 2.5 mm wide, lateral lobes ovate-triangular with sometimes falcate apex	**15**
15	Flowers wine-red; petals glabrous; column setae shortly stellate	***Telipogon jostii***
–	Flowers yellow-orange suffused with mahogany to dark vinaceous; petals pubescent on the basal half and ciliate along the margins, setae long stellate	***Telipogon marietalamae***

## Supplementary Material

XML Treatment for Telipogon marietalamae

XML Treatment for Telipogon puyensis

XML Treatment for Telipogon hystrix

## Additional information

### Artificial Intelligence (AI) use

The authors accept full responsibility for the content of the manuscript, including the disclosure of any use of AI.

Regarding the use of AI in the preparation of this manuscript, the authors declare the following: Microsoft Copilot.

**Used for**: Language, style and writing.

AI was used for English-language editing. All text was carefully reviewed and approved by the authors.

### Author ORCIDs

M.M. Jiménez https://orcid.org/0000-0002-9502-5651

N. Lapo-Gonzalez https://orcid.org/0009-0001-8863-1557

D. Gutiérrez del Pozo https://orcid.org/0000-0003-3611-7932

J.R. Kuethe https://orcid.org/0000-0001-5390-9654

L. Baquero https://orcid.org/0000-0002-1444-5727

H.X. Garzón-Suárez https://orcid.org/0000-0003-4049-1652

C. Martel https://orcid.org/0000-0001-9892-1999

G.A. Iturralde https://orcid.org/0000-0003-2456-0929

### Data availability

All of the data that support the findings of this study are available in the main text or Supplementary Information.

## Supplementary materials

Supplementary material 1 Phylogenetic trees inferred from the nrITS marker Data type pdf Explanation note **fig. S1**. Phylogenetic trees inferred from the nrITS marker. Tree on the left represents the 50% majority-rule consensus tree obtained from the Bayesian inference (BI) analysis in MrBayes (numbers at nodes represent posterior probabilities). The tree on the right represents the best-scoring maximum likelihood (ML) tree inferred with RAxML (numbers at nodes represent bootstrap support values (%) from 1,000 rapid bootstrap replicates). Scale bars represent the mean number of nucleotide substitutions per site. For *Telipogon hystrix*, the locality and field number are shown in parentheses; GB denotes GenBank. https://binary.pensoft.net/file/1816459This dataset is made available under the Open Database License (http://opendatacommons.org/licenses/odbl/1.0/). The Open Database License (ODbL) is a license agreement intended to allow users to freely share, modify, and use this Dataset while maintaining this same freedom for others, provided that the original source and author(s) are credited. Marco M. Jiménez, Nadia Lapo-Gonzalez, Diego Gutiérrez del Pozo, J. R. Kuethe, Luis Baquero, Henry X. Garzón-Suárez, Carlos Martel, Gabriel A. Iturralde

Supplementary material 2 Phylogenetic trees inferred from the *matK* marker Data type pdf Explanation note **fig. S2**. Phylogenetic trees inferred from the *matK* marker. Tree on the left represents the 50% majority-rule consensus tree obtained from the Bayesian inference (BI) analysis in MrBayes (numbers at nodes represent posterior probabilities). The tree on the right represents the best-scoring maximum likelihood (ML) tree inferred with RAxML (numbers at nodes represent bootstrap support values (%) from 1,000 rapid bootstrap replicates). Scale bars represent the mean number of nucleotide substitutions per site. For *Telipogon hystrix*, the locality and field number are shown in parentheses; GB denotes. https://binary.pensoft.net/file/1816460This dataset is made available under the Open Database License (http://opendatacommons.org/licenses/odbl/1.0/). The Open Database License (ODbL) is a license agreement intended to allow users to freely share, modify, and use this Dataset while maintaining this same freedom for others, provided that the original source and author(s) are credited. Marco M. Jiménez, Nadia Lapo-Gonzalez, Diego Gutiérrez del Pozo, J. R. Kuethe, Luis Baquero, Henry X. Garzón-Suárez, Carlos Martel, Gabriel A. Iturralde

## References

[B1] Bachman S, Moat J, Hill A, de la Torre J, Scott B (2011) Supporting Red List threat assessments with GeoCAT: geospatial conservation assessment tool. ZooKeys 150: 117–126. 10.3897/zookeys.150.2109 PMC3234434 22207809

[B2] Beentje H (2016) The Kew Plant Glossary: An Illustrated Dictionary of Plant Terms. 2^nd^ edn. Royal Botanic Gardens, Kew. Kew Publishing, Richmond, UK, 184 pp.

[B3] Bogarín D (2012) A new *Telipogon* from Mexico close to *Telipogon standleyi* (Orchidaceae: Oncidiinae). Lankesteriana 12(2): 115–119. 10.15517/lank.v0i0.11761

[B4] Braas LA, Lückel E (1982) Die Gattungen mit Bestimmungsschlüssel der *Telipogon*-Verwandtshaft (subtribus Telipogoninae Schltr.: *Telipogon* Kunth., *Trichoceros* Kunth, *Stellilabium* Schltr., *Dipterostele* Schltr., *Darwiniella* Braas & Lückel. Die Orchidee 33: 170–176.

[B5] Chase MW (2009) Subtribe Oncidiinae: *Telipogon*. In: Pridgeon AM, Cribb PJ, Chase MW, Rasmussen FN (Eds) Genera Orchidacearum, Vol. 5 Epidendroideae (Part Two), Oxford University Press Inc, New York, 362–366.

[B6] Dodson CH (2004) Native Ecuadorian Orchids, Vol. 5 (*Rodriguezia* – *Zygosepalum*). Dodson Publishing, Sarasota, Florida.

[B7] Dodson CH, Dodson PM (1980) Icones Plantarum Tropicarum: Series I, Fascicle 4. Orchids of Ecuador. Marie Selby Botanical Gardens, Sarasota, Florida.

[B8] Dodson CH, Dodson PM (1982) Icones Plantarum Tropicarum: Series I, Fascicle 5. Orchids of Ecuador. Marie Selby Botanical Gardens, Sarasota, Florida.

[B9] Dodson CH, Dodson PM (1984) Icones Plantarum Tropicarum: Series I, Fascicle 10. Orchids of Ecuador. Marie Selby Botanical Gardens, Sarasota, Florida.

[B10] Dressler RL (1999) A reconsideration of *Stellilabium* and *Dipterostele*. Harvard Papers in Botany 4(2): 469–473. http://www.jstor.org/stable/41761584

[B11] Dressler RL (2001) *Stellilabium erratum*, a comedy of blunders. Lankesteriana 2: 11–13.

[B12] Esri (2014) ArcGIS Desktop 10.3. Environmental Systems Research Institute, Redlands, California, USA. 10.15517/lank.v1i2.23144

[B13] Garay LA, Romero GA (1998) Schedulae Orchidum. Harvard Papers in Botany 3: 53–62.

[B14] Huelsenbeck JP, Ronquist F (2001) MRBAYES: Bayesian Inference of Phylogenetic Trees. Bioinformatics 17: 754–755. 10.1093/bioinformatics/17.8.754 11524383

[B15] Huera-Lucero T, Salas-Ruiz A, Changoluisa D, Bravo-Medina C (2020) Towards sustainable urban planning for Puyo (Ecuador): Amazon Forest landscape as potential green infrastructure. Sustainability12(11): 4768. 10.3390/su12114768

[B16] Iturralde GA, Monteros MF, Jiménez MM, Martel C, Baquero LE (2023) *Telipogon pillaropatatensis* (Oncidiinae): a new species from the East-Central Andes of Ecuador. Lankesteriana 23(3): 495–509. 10.15517/lank.v23i3.58100

[B17] Iturralde GA, Jiménez MM, Martel C, Baquero L, Restrepo E, Rodríguez-Prieto A, Monteros M, Jost L (2024) *Telipogon nigropurpureus* (Orchidaceae: Oncidiinae): new insights on its morphology and distribution in Colombia and Ecuador. Phytotaxa 647(2): 189–198. 10.11646/phytotaxa.647.2.5

[B18] IUCN (2024) Guidelines for Using the IUCN Red List Categories and Criteria. Version 16. Prepared by the Standards and Petitions Committee. https://www.iucnredlist.org/documents/RedListGuidelines.pdf

[B19] Jiménez MM, Martel C, Garzón-Suárez HX, Baquero LE, Mashendo V, Iturralde GA (2024) *Telipogon leisberthvelezii* (Orchidaceae: Oncidiinae), a new orchid species from the Cordillera del Cóndor in Ecuador. Kew Bulletin 79(1): 97–106. 10.1007/s12225-023-10145-5

[B20] Jiménez MM, Baquero L, Garzón-Suárez HX, Martel C, Chamaya JA, Lapo-González N, Werner FA, Iturralde GA (2025) *Telipogon villonacoensis* (Orchidaceae: Oncidiinae), a new species with an unexpectedly wide distribution from the northern and central Andes of South America. Plant Ecology and Evolution 158(3): 392–402. 10.5091/plecevo.157554

[B21] Kasajima I, Ide Y, Ohkama-Ohtsu N, Hayashi H, Yoneyama T, Fujiwara T (2004) A protocol for rapid DNA extraction from *Arabidopsis thaliana* for PCR analysis. Plant Molecular Biology Reporter 22(1): 49–52. 10.1007/BF02773348

[B22] Kolanowska M, Trejo RM, Lipińska M (2017) A new species of *Stellilabium* (Orchidaceae) segregated from *S. morganiae*. Polish Botanical Journal 62(1): 21–26. 10.1515/pbj-2017-0002

[B23] MAE [Ministerio de Ambiente y Energía] (2026) Mapa interactivo. Ministerio del Ambiente y Energía. http://ide.ambiente.gob.ec/mapainteractivo/ [Accessed 30.03.2026]

[B24] Martel C (2016) Nomenclatural changes in species of *Telipogon* (Orchidaceae) from Ecuador and Bolivia. Orquideología 33(1): 38–40.

[B25] Martel C, Collantes B, Egoavil L (2017) *Telipogon huancavelicanus* sp. nov. (Orchidaceae) from Peru, and an updated description of *T. deuterocuscoensis*. Nordic Journal of Botany 35(5): 539–545. 10.1111/njb.01520

[B26] Martel C, Neubig KM, Williams NH, Ayasse M (2020) The uncinate viscidium and floral setae, an evolutionary innovation and exaptation to increase pollination success in the *Telipogon* alliance (Orchidaceae: Oncidiinae). Organisms Diversity & Evolution 20(3): 537–550. 10.1007/s13127-020-00457-w

[B27] Martel C, Hernández AG, Iturralde GA, Collantes B (2025) *Telipogon rojasiae* (Orchidaceae, Oncidiinae), a new species from relict forests on the western slopes of the northern Peruvian Andes. PhytoKeys 265: 49–60. 10.3897/phytokeys.265.164394 PMC12550512 41142440

[B28] Ministerio del Ambiente [del Ecuador] (2013) Sistema de Clasificación de los Ecosistemas del Ecuador Continental de los Andes. Quito, Ecuador.

[B29] Neill DA, Asanza M (2014) *Browneopsis puyensis* (Leguminosae: Caesalpinioideae: Detarieae), a new species from Amazonian Ecuador. Journal of the Botanical Research Institute of Texas 8(2): 511–516.

[B30] Neubig KM, Whitten WM, Williams NH, Blanco MA, Endara L, Burleigh JG, Silvera K, Cushman J, Chase M (2012) Generic recircumscriptions of Oncidiinae (Orchidaceae: Cymbidieae) based on maximum likelihood analysis of combined DNA datasets. Botanical Journal of the Linnean Society 168(2): 117–146. 10.1111/j.1095-8339.2011.01194.x

[B31] POWO (2026) Plants of the World Online. Facilitated by the Royal Botanic Gardens, Kew. http://www.plantsoftheworldonline.org [Accessed 15 June 2025]

[B32] Pupulin F (2022) Vanishing Beauty: Native Costa Rica Orchids. Vol. 3. *Restrepia*-*Zootrophion* and appendices. Koeltz Botanical Books, Oberreifenberg, 467 pp.

[B33] Reichenbach HG (1858) Xenia orchidacea: Beiträge zur Kenntniss der Orchideen (Vol. 1). F.A. Brockhaus. 10.5962/bhl.title.462

[B34] Schlechter R (1915) Die Orchideen: ihre Beschreibung, Kultur und Züchtung. Handbuch für Orchideenliebhaber, Züchter und Botaniker. P. Parey, Berlin, 837 pp. https://www.biodiversitylibrary.org/item/100868

[B35] Schlechter R (1921) Die Orchideenfloren der südamerikanischen Kordillerenstaaten, III. Ecuador. Repertorium Specierum Novarum. Regni Vegetabilis, Beihefte 8: 1–172. https://www.biodiversitylibrary.org/item/112026

[B36] Stamatakis A (2014) RAxML version 8: a tool for phylogenetic analysis and post-analysis of large phylogenies. Bioinformatics 30(9): 1312–1313. 10.1093/bioinformatics/btu033 PMC3998144 24451623

[B37] Szlachetko DL, Margońska HB (2006) *Stellilabium amicorum* Szlach. & Marg., spec. nov. (Orchidaceae, Telipogoneae), eine neue Art aus Ecuador. Die Orchidee 57(3): 320–324.

[B38] Tamura K, Stecher G, Kumar S (2021) MEGA11: Molecular Evolutionary Genetics Analysis version 11. Molecular Biology and Evolution 38(7): 3022–3027. 10.1093/molbev/msab120 PMC8233496 33892491

[B39] Williams NH, Whitten WM, Dressler RL (2005) Molecular systematics of *Telipogon* (Orchidaceae: Oncidiinae) and its allies: nuclear and plastid DNA sequence data. Lankesteriana 5(3): 163–184. 10.15517/lank.v5i3.19754

[B40] Zambrano BJ, Bogarin D, Solano R (2018) *Telipogon sonia-juaniorum* (Orchidaceae: Oncidiinae) a new species from Southwestern Ecuador. Phytotaxa 340(2): 167–174. 10.11646/phytotaxa.340.2.5

