# Supplementary material for: Telipogon marietalamae and T. puyensis (Orchidaceae, Oncidiinae), two new species from the Amazonian region of Ecuador
Source: PhytoKeys. 2026 Oct 22;280:213–38. doi: 10.3897/phytokeys.280.196965 (PMC13720663; doi:10.3897/phytokeys.280.196965)
Supplement: Supplementary file 1 [file phytokeys-280-213_article-196965__-s001.pdf]

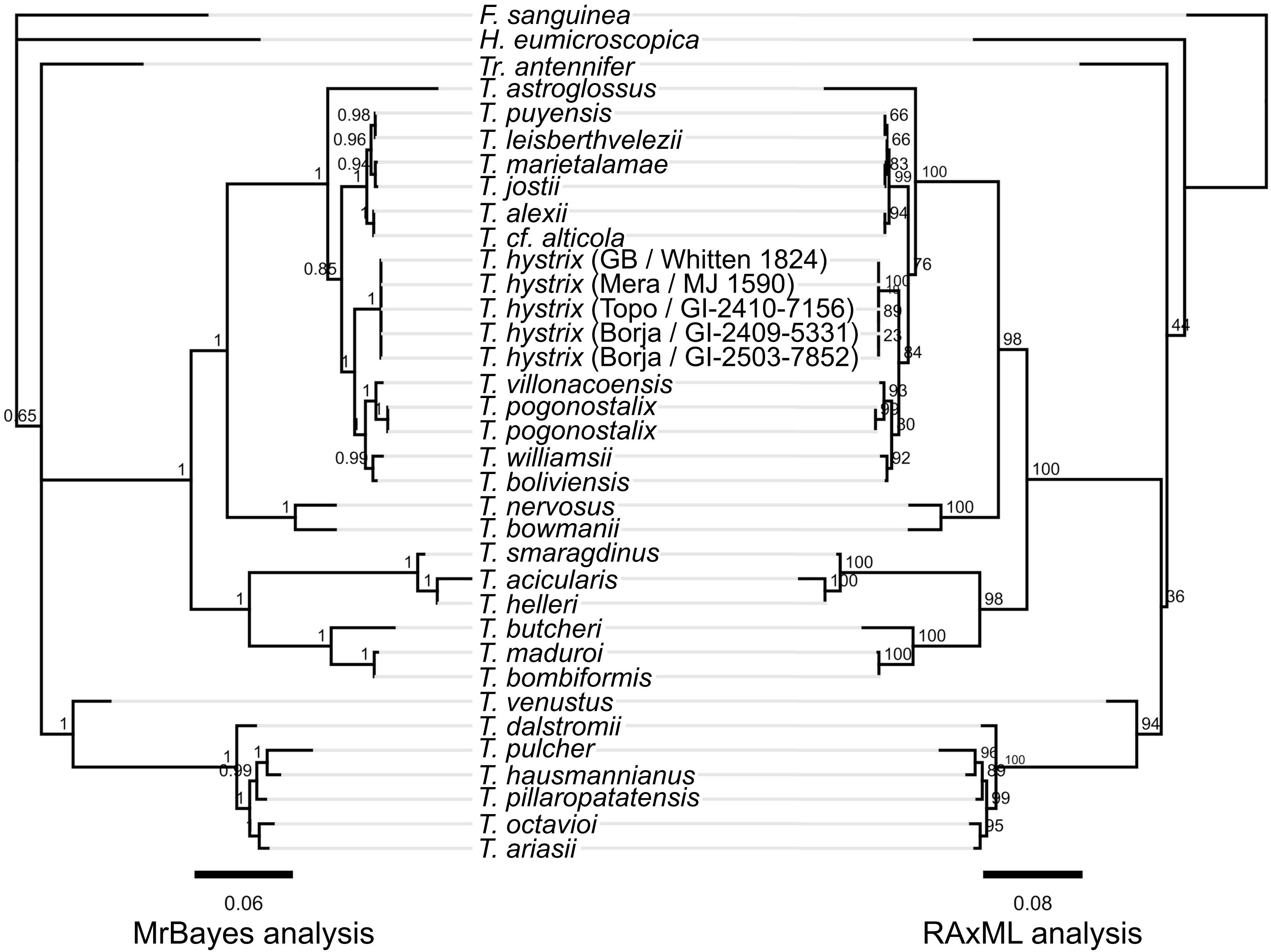
F. sanguinea
H. eumicroscopica
Tr. antennifer
T. astroglossus
T. puyensis
T. leisberthvelezii
T. marietalamae
T. jostii
T. alexii
T. cf. alticola
T. hystrix (GB / Whitten 1824)
T. hystrix (Mera / MJ 1590)
T. hystrix (Topo / GI-2410-7156)
T. hystrix (Borja / GI-2409-5331)
T. hystrix (Borja / GI-2503-7852)
T. villonacoensis
T. pogonostalix
T. pogonostalix
T. williamsii
T. boliviensis
T. nervosus
T. bowmanii
T. smaragdinus
T. acicularis
T. helleri
T. butcheri
T. maduroi
T. bombiformis
T. venustus
T. dalstromii
T. pulcher
T. hausmannianus
T. pillaropatatensis
T. octavioi
T. ariasii
0.06
MrBayes analysis
0.08
RAxML analysis
